# Evaluation of Early and Delayed Meloxicam Treatment Against Regulated Cell Death Pathways and ERK1/2 Phosphorylation in a Rat Model of Renal Ischemia–Reperfusion Injury

**DOI:** 10.3390/biomedicines14081760

**Published:** 2026-08-05

**Authors:** Mahmut Şahin, Hasan Başçil, Alper Serhat Kumru, Mustafa Özkaraca

**Affiliations:** 1 Department of Pharmacology and Toxicology, Faculty of Veterinary Medicine, Sivas Cumhuriyet University, 58140 Sivas, Türkiye; askumru@cumhuriyet.edu.tr; 2 Department of Cardiovascular Surgery, Faculty of Medicine, Sivas Cumhuriyet University, 58140 Sivas, Türkiye; hbascil@cumhuriyet.edu.tr; 3 Department of Pathology, Faculty of Veterinary Medicine, Sivas Cumhuriyet University, 58140 Sivas, Türkiye; mustafaozkaraca@cumhuriyet.edu.tr

**Keywords:** renal ischemia–reperfusion, meloxicam, necroptosis, MAPK, KIM-1, cystatin C, acute kidney injury

## Abstract

**Objectives**: Renal ischemia–reperfusion (I/R) injury is one of the most important pathological triggers of acute kidney injury. This study aimed to investigate the protective effects of meloxicam, a selective cyclooxygenase-2 (COX-2) inhibitor, against renal I/R injury through specific cell death pathways including inflammation, apoptosis, necroptosis, and the MAPK/ERK pathway, which is potentially linked to regulated cell death mechanisms such as ferroptosis. **Methods**: Male Wistar Albino rats weighing 280–300 g were used in the study and were divided into four groups: Sham, IR (40 min ischemia + 120 min reperfusion), Meloxicam + IR, and Meloxicam + IR1. Bilateral renal ischemia was induced for 40 min via a retroperitoneal approach under anesthesia. Meloxicam was administered intravenously at a dose of 1 mg/kg at the initiation of reperfusion in the Meloxicam + IR group, whereas in the Meloxicam + IR1 group, the same dose was administered 1 h after the onset of reperfusion. Total reperfusion time was 120 min in both groups. Renal function parameters (BUN and creatinine) and oxidative stress markers (TAS and TOS) were measured. Inflammatory cytokines (IL-6, IL-1β, and IL-10), the glomerular filtration injury marker Cystatin C, the tubular injury marker KIM-1, the apoptotic marker Caspase 3, the necroptosis markers RIPK3 and MLKL, and MAPK signaling pathway alterations (ERK1/2 and pERK1/2 levels) associated with cellular survival and death signaling were evaluated. **Results**: Most notably, meloxicam markedly modulated apoptosis, the expression of necroptosis markers RIPK3 and MLKL, and the activation of pERK1/2, a key node in MAPK signaling that is regulatory in cell survival and cell death processes. The drug also suppressed pro-inflammatory cytokines (IL-6 and IL-1β) while preserving anti-inflammatory IL-10 levels. Furthermore, improvements were observed in the levels of KIM-1, a marker of tubular injury, and Cystatin C, a marker of glomerular filtration impairment. Consequently, meloxicam administration significantly reduced the elevated serum creatinine and TOS levels observed in the IR group, although serum BUN levels remained without notable alteration. **Conclusions**: The findings of this study suggest that meloxicam may extend beyond its role as a classical anti-inflammatory agent, potentially offering biochemical and functional protection against renal I/R injury in association with the modulation of specific cell death mechanisms, including necroptosis and apoptosis, as well as the MAPK signaling pathway.

## 1. Introduction

Acute Kidney Injury (AKI) is among the most significant clinical conditions associated with increased morbidity and mortality in hospitalized patients [1,2]. One of the principal mechanisms underlying this condition is renal ischemia–reperfusion (I/R) injury, which is characterized by a temporary interruption of renal blood flow followed by restoration of perfusion [3,4]. Renal I/R injury is an unavoidable phenomenon encountered during major abdominal surgeries, renal transplantation, cardiovascular bypass procedures, and septic shock [5,6].

Regulated cell death (RCD) pathways play a pivotal role in the pathogenesis of renal I/R injury. Historically studied in isolation, apoptosis, necroptosis, and ferroptosis are now recognized to engage in a highly intricate molecular crosstalk. Ferroptosis is classically defined as an iron-dependent form of non-apoptotic RCD, characterized primarily by the accumulation of lethal lipid reactive oxygen species (ROS), depletion of glutathione, and loss of activity of the lipid repair enzyme glutathione peroxidase 4 [7]. While the mitogen-activated protein kinase (MAPK) pathway, specifically the phosphorylation of extracellular signal regulated kinase 1/2 (p-ERK1/2), is not a primary defining feature of ferroptosis, it has been shown to act as a crucial upstream modulator that can promote or amplify ferroptotic cascades under oxidative stress conditions [8]. During renal I/R, the depletion of cellular energy and surge in ROS trigger a synchronized demise: apoptotic pathways (mediated by Caspase 3) coexist and cross-regulate necroptotic machinery (driven by the RIPK1/RIPK3/MLKL axis), while simultaneously predisposing cells to ferroptotic execution due to disrupted intracellular iron and lipid homeostasis [9,10].

Renal ischemia begins with the interruption of oxygen and nutrient delivery to tubular epithelial cells, which have a high metabolic demand [11]. During the ischemic period, intracellular ATP stores are rapidly depleted, intracellular acidosis develops due to the shift toward anaerobic glycolysis, and calcium overload occurs as a result of impaired sodium potassium pump (Na^+^/K^+^-ATPase) function [12,13]. However, the most destructive phase of tissue injury paradoxically occurs during reperfusion, when blood flow is restored [14,15].

During reperfusion, the sudden influx of oxygen into the tissue results in massive production of reactive oxygen species (ROS) due to dysfunction of the mitochondrial electron transport chain [16]. This increase in ROS levels triggers lipid peroxidation, disrupts cellular membrane integrity, and causes nuclear DNA damage [17]. Simultaneously, activation of pro-inflammatory signaling pathways initiates neutrophil and macrophage infiltration into the tissue, leading to a robust inflammatory response characterized by elevated levels of IL-6, IL-1β, and TNF-α [18]. Numerous previous studies have demonstrated that this process is not merely a consequence of random cell death but rather progresses through regulated cell death pathways. These pathways include necroptosis mediated by RIPK3 and MLKL, apoptosis driven by caspase-3 activation, and regulated cell death processes potentially associated with MAPK/ERK signaling cascades [19,20,21].

Meloxicam is a non-steroidal anti-inflammatory drug (NSAID) belonging to the oxicam class and exhibiting high selectivity toward the cyclooxygenase-2 (COX-2) enzyme [22]. It is widely used in clinical practice for the treatment of chronic inflammatory conditions such as rheumatoid arthritis and osteoarthritis due to its analgesic and anti-inflammatory properties [23]. The primary mechanism of action of meloxicam is associated with inhibition of the COX-2 enzyme, which catalyzes the conversion of arachidonic acid into pro-inflammatory prostaglandins [24]. Unlike conventional NSAID, the COX-2 selectivity of meloxicam contributes to a lower incidence of gastric and renal adverse effects [25]. However, recent studies suggest that meloxicam may possess pleiotropic properties beyond COX-2 inhibition, including suppression of oxidative stress, anti-apoptotic activity, and promotion of tissue repair [26,27,28]. Despite the well-known anti-inflammatory and anti-apoptotic properties of the selective COX-2 inhibitor meloxicam, a significant knowledge gap remains regarding whether meloxicam can concurrently modulate this triad of RCD pathways in a time dependent manner during renal reperfusion [29].

**Figure 1:**
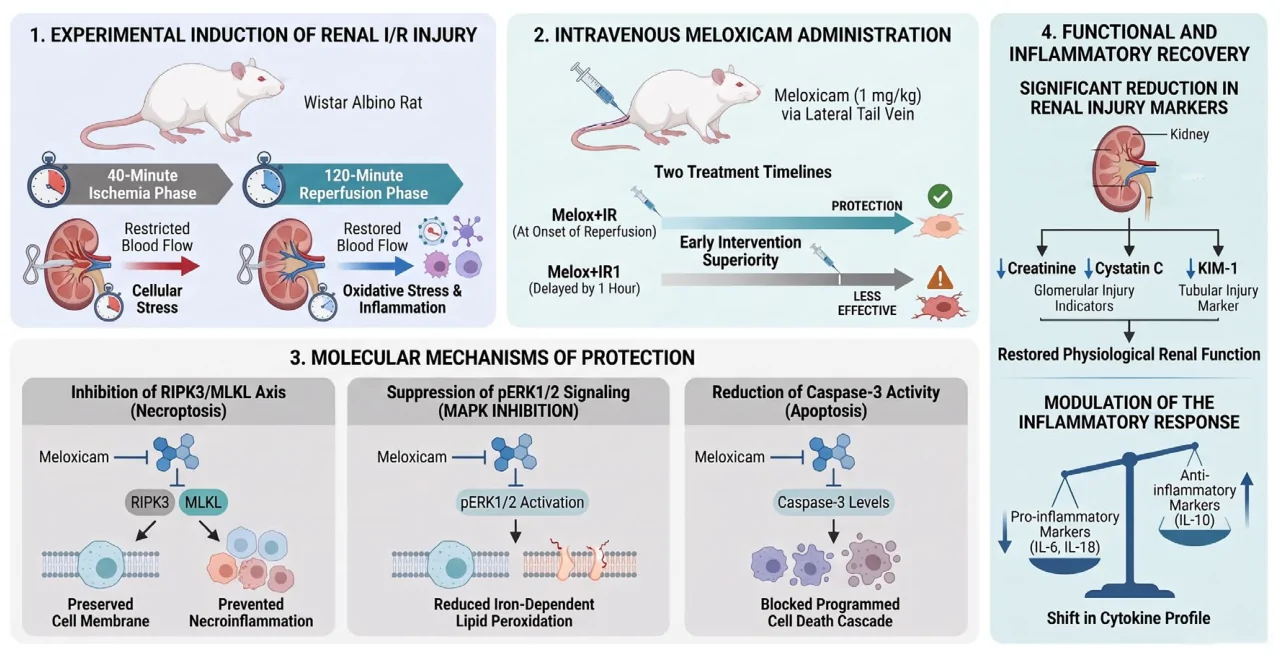
Experimental design of meloxicam treatment groups and graphical abstract.

Although several studies have proposed that meloxicam may protect tubular epithelial cells by suppressing the inflammatory cascade in renal I/R models [30,31,32], no study has comprehensively evaluated its apoptotic, necroptotic, and ferroptotic effects simultaneously. In light of the current literature, it is hypothesized that the administration of meloxicam during the early phase of renal I/R injury may modulate the inflammatory response and suppress specific cell death pathways [30,32,33,34]. Therefore, the present study aimed to fill this gap in the literature by comprehensively analyzing the renoprotective effects of meloxicam at molecular and histopathological levels through the evaluation of tubular injury, inflammation, apoptosis, necroptosis, and regulated cell death pathways associated with MAPK/ERK signaling (Figure 1).

## 2. Materials and Methods

### 2.1. Experimental Model

This study was conducted with the approval of the Sivas Cumhuriyet University Local Ethics Committee for Animal Experiments (Approval No. 98, dated 6 November 2025). Healthy male Wistar Albino rats weighing 280–300 g were used in the experiments. Animals were housed under controlled environmental conditions (22 ± 2 °C temperature and 50–60% humidity) with a 12 h light/12 h dark cycle and were provided ad libitum access to standard laboratory chow and drinking water. Prior to the experimental procedures, the animals were fasted for 12 h while water was provided without restriction. The rats were randomly assigned to the following four groups:

Sham Group (*n = 6*): Only a retroperitoneal surgical incision was performed, and the bilateral renal pedicles were exposed without inducing ischemia.

Renal Ischemia–Reperfusion (IR) Group (*n = 6*): Bilateral renal ischemia was induced for 40 min followed by 120 min of reperfusion.

Meloxicam + IR Group (0 h Reperfusion) (*n = 6*): Following 40 min of ischemia, 1 mg/kg meloxicam was administered intravenously at the onset of reperfusion, and reperfusion was continued for 120 min.

Meloxicam + IR1 Group (1st-Hour Reperfusion) (*n = 6*): Following 40 min of ischemia, reperfusion was initiated and maintained for 120 min. No treatment was administered during the first 60 min of reperfusion. At the 61st minute of reperfusion, 1 mg/kg meloxicam was administered intravenously, and reperfusion was continued for an additional 60 min.

All animals were anesthetized using a ketamine–xylazine combination (90 mg/kg ketamine and 9 mg/kg xylazine, intraperitoneally). Adequate anesthetic depth was confirmed by the absence of the pedal withdrawal reflex. During the surgical procedures, the core body temperature of the rats was monitored rectally and strictly maintained within the physiological range of 37.0 ± 0.5 °C. To compensate for anesthesia-induced heat loss, this was achieved by placing the animals on a temperature-controlled heating pad set at 40 °C. The animals were placed in the prone position, and the bilateral hypochondriac regions were shaved and disinfected with 10% povidone-iodine solution. A dorsolateral hypochondriac incision approximately 1 cm in length was made parallel to the midline, and the retroperitoneal space was accessed. This approach was preferred to minimize manipulation of intra-abdominal organs and reduce surgical trauma.

**Figure 2:**
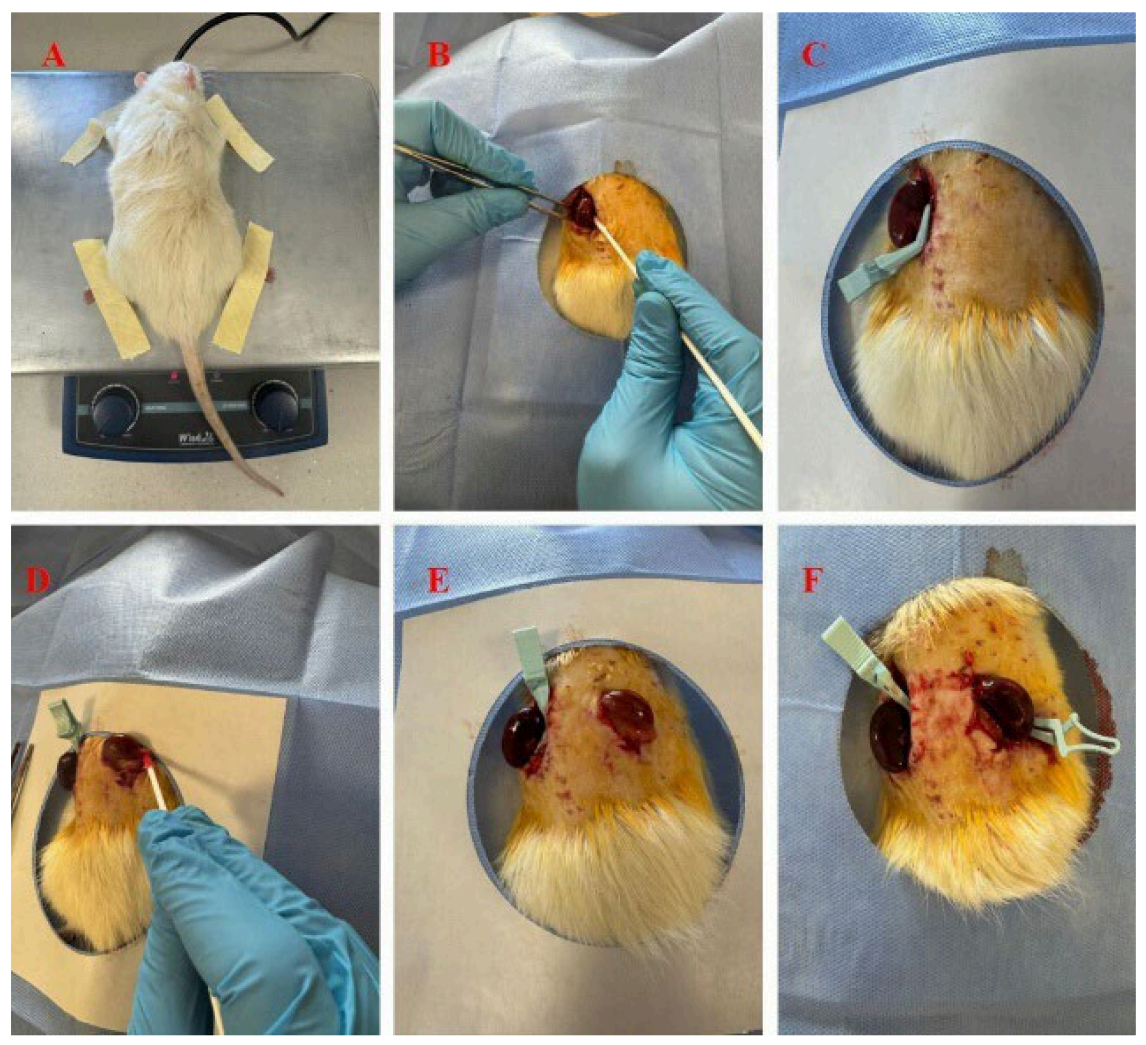
(**A**) Positioning of the experimental animal on the temperature-controlled platform. (**B**) Exposure of the left kidney via a retroperitoneal approach. (**C**) Placement of a micro-bulldog clamp on the left renal pedicle to induce ischemia. (**D**) Exposure of the right kidney. (**E**) Left kidney under ischemia and right kidney before clamp application. (**F**) Bilaterally ischemic kidneys.

Following blunt dissection, the renal fascia was carefully separated, and the renal pedicles were exposed while preserving the adrenal glands. Bilateral renal ischemia was induced by applying miniature bulldog clamps to both renal pedicles for 40 min. Ischemia was visually confirmed by the characteristic discoloration (pallor/cyanosis) of the kidneys (Figure 2).

Immediately before clamp removal at the end of the ischemic period, rats in the Melox + IR (0 h reperfusion) group received meloxicam at a dose of 1 mg/kg dissolved in 0.5 mL physiological saline through the lateral tail vein (*v. caudalis lateralis*). The dose of meloxicam (1 mg/kg) was selected based on previous literature demonstrating its selective COX-2 inhibitory efficacy and renal protective properties in rodent models [32]. This dose was chosen to provide effective COX-2 inhibition and anti-inflammatory activity while avoiding the non-selective COX-1 inhibition, altered renal perfusion, and potential nephrotoxicity associated with higher doses of meloxicam. Following meloxicam administration, the clamps were removed to initiate reperfusion. To ensure strict methodological standardization and control for injection induced physiological stress, animals in both the Sham and untreated IR groups received an equivalent volume of sterile physiological saline via the same administration route at the corresponding time points. For the Melox + IR1 group, the clamps were removed immediately at the end of the ischemic period to initiate reperfusion without any prior drug administration; these rats received the same dose of meloxicam (1 mg/kg, i.v.) 1 h after the onset of reperfusion. In all groups, restoration of the normal renal color was visually confirmed, after which the kidneys were returned to their anatomical positions and the surgical wounds were temporarily closed with simple sutures to prevent dehydration during the reperfusion period. The animals were randomly assigned to the experimental groups prior to any surgical interventions. During the entire course of the study, no animal mortality or unexpected complications occurred, and no animals or data points were excluded from the study. Consequently, all experimental procedures and subsequent analyses were completed with the initial sample size of *n = 6* animals per group.

### 2.2. Biochemical Analyses

At the end of the designated reperfusion periods, blood samples were collected by intracardiac puncture, and the animals were subsequently sacrificed by exsanguination. Serum blood urea nitrogen (BUN) and creatinine concentrations were measured using an automated biochemical analyzer (BIS Systems, BA2000 Led Tech., Barcelona, Spain) to evaluate biochemical alterations associated with the loss of glomerular function.

Serum Total Antioxidant Status (TAS) and Total Oxidant Status (TOS) levels were determined using commercially available Rel Assay diagnostics kits (Rel Assay Diagnostics, TAS catalog no: RL0017, TOS catalog no: RL0024, Gaziantep, Turkey). These measurements were performed using automated colorimetric/spectrophotometric methods developed by Erel. The TAS assay is based on the bleaching of the characteristic color of a more stable ABTS (2,2′-azino-bis(3-ethylbenzothiazoline-6-sulfonic acid)) radical cation by antioxidants, and the results are expressed as mmol Trolox Equivalent/L. The TOS assay is based on the oxidation of ferrous ion to ferric ion in the presence of various oxidant species in an acidic medium, and the results are expressed as μmol H_2_O_2_ Equivalent/L. The absorbance changes were measured spectrophotometrically at the wavelengths specified in the manufacturer’s instructions (Epoch Microplate Reader, Agilent Technologies, Gen-5 software, Winooski, VT, USA).

To eliminate potential investigator bias, all biochemical analyses were performed in a blinded manner. All serum and tissue samples were coded with anonymous identification numbers by an independent researcher, and the laboratory investigators conducting the spectrophotometric and colorimetric assays remained completely blinded to the experimental group allocations until the final data processing and statistical analysis.

### 2.3. Histopathology

For the assessment of renal tissue injury, kidney specimens were fixed in 10% neutral-buffered formalin solution. Following routine alcohol–xylene tissue processing, the samples were embedded in paraffin blocks. Sections of 5 μm thickness were prepared and stained with hematoxylin and eosin (H&E). Six randomly selected microscopic fields from each specimen were evaluated semi-quantitatively for tubular necrosis, tubular degeneration, and interstitial hemorrhage using the following scoring system: absent (0), mild (1), moderate (2), and severe (3). All histopathological evaluations were performed by an experienced pathologist who was completely blinded to the experimental groups and treatment protocols. To prevent observer bias, all tissue slides were randomized and assigned unique, anonymous identification codes prior to light microscopic examination.

### 2.4. Immunohistochemistry and Immunofluorescence Examination

Kidney tissues were examined immunohistochemically to evaluate tubular degeneration, necroptosis, and apoptosis. Following the blocking procedure, tissue sections were incubated overnight at +4 °C with primary antibodies diluted at 1:200. As the secondary detection system, the Large Volume Detection System: anti-Polyvalent, HRP (Thermo Fisher Scientific, Fremont, CA, USA; Cat. No. TP-125-HL) was used according to the manufacturer’s instructions. DAB (3,3′-Diaminobenzidine) was employed as the chromogen. After counterstaining with Mayer’s hematoxylin, immunopositive reactions in six randomly selected fields were evaluated semi-quantitatively.

In the immunohistochemical analyses, KIM-1 (Kidney Injury Molecule-1) was used to assess the degree of tubular degeneration, Caspase-3 was used as an indicator of apoptosis, and RIPK3 (Receptor-Interacting Protein Kinase-3) together with MLKL (Mixed Lineage Kinase Domain-Like Pseudokinase) were used to evaluate necroptosis (Table 1).

For double-merged immunofluorescence analyses, ERK1/2 and pERK1/2 were used to evaluate MAPK signal pathway, IL-6 and IL-1β were used as markers of cellular inflammation, IL-10 was used to assess anti-inflammatory activity, and Cystatin C was employed as a marker of glomerular filtration injury. IL-1β, IL-10, and ERK1/2 immunopositivities were visualized as green fluorescence, whereas IL-6, Cystatin C, and pERK1/2 immunopositivities were visualized as red fluorescence (Table 1).

Double-merged immunofluorescence and immunohistochemical evaluations were performed in a double-blind manner. Positive staining in six randomly selected microscopic fields was examined using a fluorescence-equipped light microscopy system (Zeiss Axiolab 5, Axiocam 305 Color Camera, Colibri 3, Oberkochen, Germany). Immunopositivity was analyzed using Zen Blue 3.1 software (Zeiss, Oberkochen, Germany) and graded semi-quantitatively as follows: Grade 0 (absent), Grade 1 (mild), Grade 2 (moderate), Grade 3 (severe), and Grade 4 (very severe). The semi-quantitative scoring of immunofluorescence staining intensity was conducted in a strictly blinded manner. The evaluating histopathologist examined the randomized and coded slides without any prior knowledge of the treatment design.

### 2.5. Statistical Analysis

Statistical analyses were performed using GraphPad Prism software (Version 8.0.1). Data normality was evaluated using the Shapiro–Wilk test, and the homogeneity of variances was verified via Levene’s test. Quantitative continuous variables satisfying parametric assumptions were analyzed using One-way Analysis of Variance (ANOVA), followed by Tukey’s post hoc test for multiple pairwise comparisons between groups. Non-parametric data, including semiquantitative histopathological and immunohistochemical scores, were evaluated using the Kruskal–Wallis test followed by Dunn’s post hoc test. The sample size of *n = 6* animals per group was determined based on well-established similar experimental models in the literature and aligned with institutional ethical guidelines aiming to minimize animal usage while maintaining statistical validity. *p* value of less than 0.05 was considered statistically significant.

## 3. Results

### 3.1. Histopathological Findings

**Figure 3:**
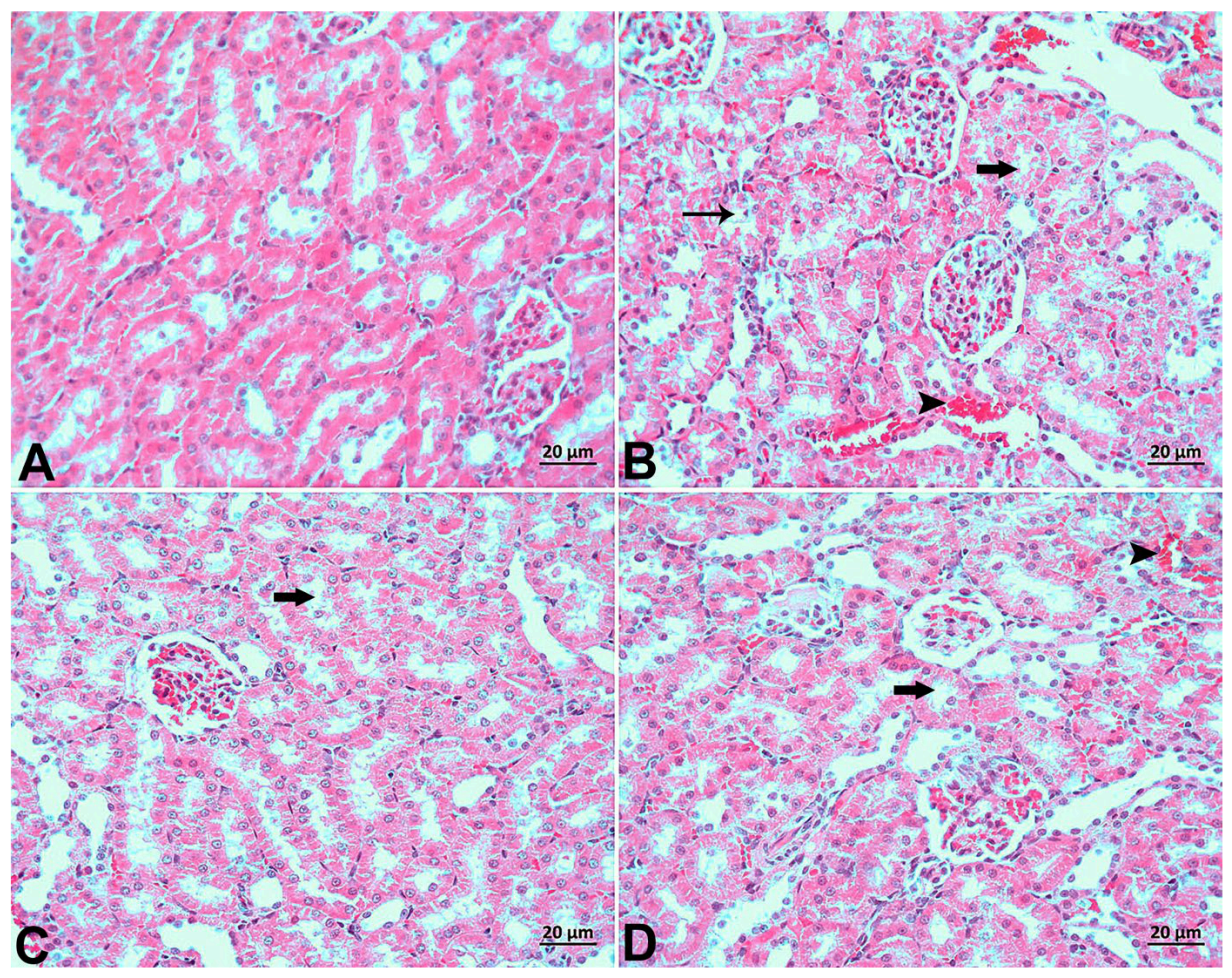
Hematoxylin and Eosin (H&E) examination. (**A**) Sham group: Normal histological appearance. (**B**) IR group: Severe necrotic tubules (thin arrow), degenerated tubules (arrow), and moderate interstitial hemorrhage (arrowhead). (**C**) Melox + IR group: Mild necrotic and degenerated tubules (arrow). (**D**) Melox + IR1 group: Moderate degenerated tubules (arrow) and mild interstitial hemorrhage (arrowhead).

**Figure 4:**
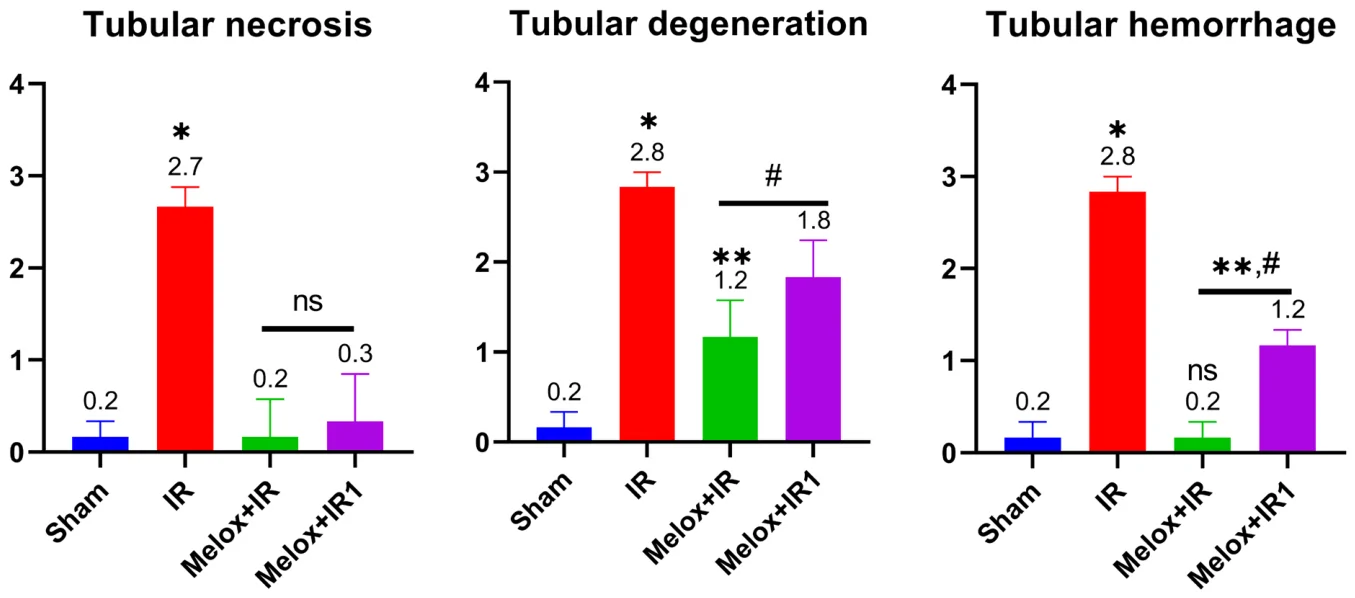
Effects of early and delayed meloxicam treatment on tubular injury levels. Data are presented as median range with individual data points plotted (*n = 6* per group). * *p* < 0.05; ** *p* < 0.01 compared to the Sham group; # *p* < 0.05 compared to the IR group (Kruskal–Wallis followed by Dunn’s post hoc test). ns, not significant.

While no histopathological alterations were detected in the Sham group, statistically significant histopathological differences were observed in all other experimental groups. The IR group exhibited severe tubular epithelial cell necrosis and degeneration, accompanied by moderate interstitial hemorrhage. In the Melox + IR1 group, tubular necrosis was absent, while tubular degeneration was moderate and interstitial hemorrhage was mild. The group presenting the lowest histopathological damage score was Melox + IR, exhibiting only mild tubular degeneration (Figure 3). Compared to the untreated I/R group, tubular necrosis, tubular degeneration, and tubular hemorrhage scores decreased by 13.50-fold, 2.33-fold, and 2.80-fold, respectively, in the Melox + IR group. In the Melox + IR1 group, these reduction rates were recorded as 1.35-fold, 1.55-fold, and 2.33-fold, respectively (Figure 4).

### 3.2. Biochemical Findings

The impact of renal functional changes on blood serum was evaluated via alterations in serum BUN and Creatinine levels. In all experimental groups, BUN values increased significantly compared to the Sham group. Conversely, serum creatinine levels in the meloxicam-treated groups demonstrated a significant decline compared to the IR group, approaching baseline levels of the Sham group. The most pronounced improvement in serum creatinine levels was observed in the Melox + IR group. Although BUN levels did not show significant alterations in our study, creatinine levels exhibited a 32.96% and 21.97% reduction in the Melox + IR and Melox + IR1 groups, respectively, compared to the untreated I/R group.

Specifically, compared to the untreated I/R group, meloxicam administration in the Melox-IR group was associated with a 28.14% reduction in serum TAS levels, whereas a minimal decrease of only 0.60% was observed in the Melox-IR1 group. Conversely, serum TOS levels demonstrated corresponding increases of 28.85% and 3.73% in the Melox-IR and Melox-IR1 groups, respectively, when compared to the I/R group.

### 3.3. Immunohistochemistry and Immunofluorescence Examination Findings

In our study, compared to the untreated I/R group, levels of the pro-inflammatory cytokine IL-1β demonstrated a 39.28% reduction in both the Melox + IR and Melox + IR1 groups. Regarding IL-6 levels, a 57.14% reduction was observed in the Melox + IR group, whereas the decrease in the Melox + IR1 group was recorded at 28.50%. In terms of the anti-inflammatory cytokine IL-10, compared to the control group, a 1.83-fold decrease was observed in the Melox + IR group, while this reduction rate was recorded as 4.12-fold in the Melox + IR1 group. Regarding Cystatin C levels, a significant 1.83-fold reduction was recorded in the Melox + IR group compared to the IR group, whereas no such significant alteration was observed in the Melox + IR1 group.

In our study, a significant downregulation in necroptosis-related RIPK3 and MLKL immunoreactivity was observed in the meloxicam-treated groups compared to the IR group, with this reduction being notably more pronounced in the Melox + IR group. Specifically, while no substantial changes in MLKL levels were detected in the Melox + IR1 group, a significant 2.12-fold decrease was recorded in the Melox + IR group compared to the IR group. Regarding RIPK3 levels, meloxicam intervention in the Melox + IR and Melox + IR1 groups yielded corresponding reductions of 3.37-fold and 1.58-fold, respectively, compared to the untreated IR group. These findings highlight that the modulation of necroptotic pathways is highly sensitive to the timing of meloxicam administration, showing a far more potent regulatory effect when administered early.

In this study, total ERK1/2 and its phosphorylated active isoform, p-ERK1/2, were demonstrated via the double immunofluorescence method. Regarding total ERK1/2 immunoreactivity, no substantial differences were recorded in the meloxicam administered groups compared to the IR group. However, the phosphorylation of ERK1/2 was suppressed in the meloxicam-treated groups, leading to a significant decrease in active pERK1/2 levels. Specifically, compared to the untreated IR group, p-ERK1/2 levels demonstrated a 3.50-fold reduction in the Melox + IR group, whereas a 1.38-fold decrease was recorded in the Melox + IR1 group.

Immunohistochemical evaluations of KIM-1 and Caspase 3 demonstrated that both parameters were statistically significantly reduced in the meloxicam treatment groups compared to the IR group, KIM-1 levels demonstrated a 33% reduction in the Melox + IR group, whereas a decrease of only 5.5% was recorded in the Melox + IR1 group. Regarding Caspase 3 semi-quantitative evaluations, levels in the Melox + IR and Melox + IR1 groups were 3.5-fold and 1.55-fold lower, respectively, than those in the IR group. Strikingly, early meloxicam administration in the Melox + IR group suppressed Caspase 3 to levels closely resembling the Sham group, underscoring its potent anti-apoptotic association when administered without delay.

## 4. Discussion

The data obtained from this study suggest that meloxicam administration during the acute phase of renal I/R injury acts not merely as a conventional anti-inflammatory agent, but also as a powerful modulator of specific cell death pathways such as necroptosis, ferroptosis, and apoptosis. While the 40 min ischemia and subsequent reperfusion period inflicted both functional and structural damage on the kidney tissue, meloxicam’s intervention provided multifaceted protection.

### 4.1. Discussion of Biochemical Data

**Figure 5:**
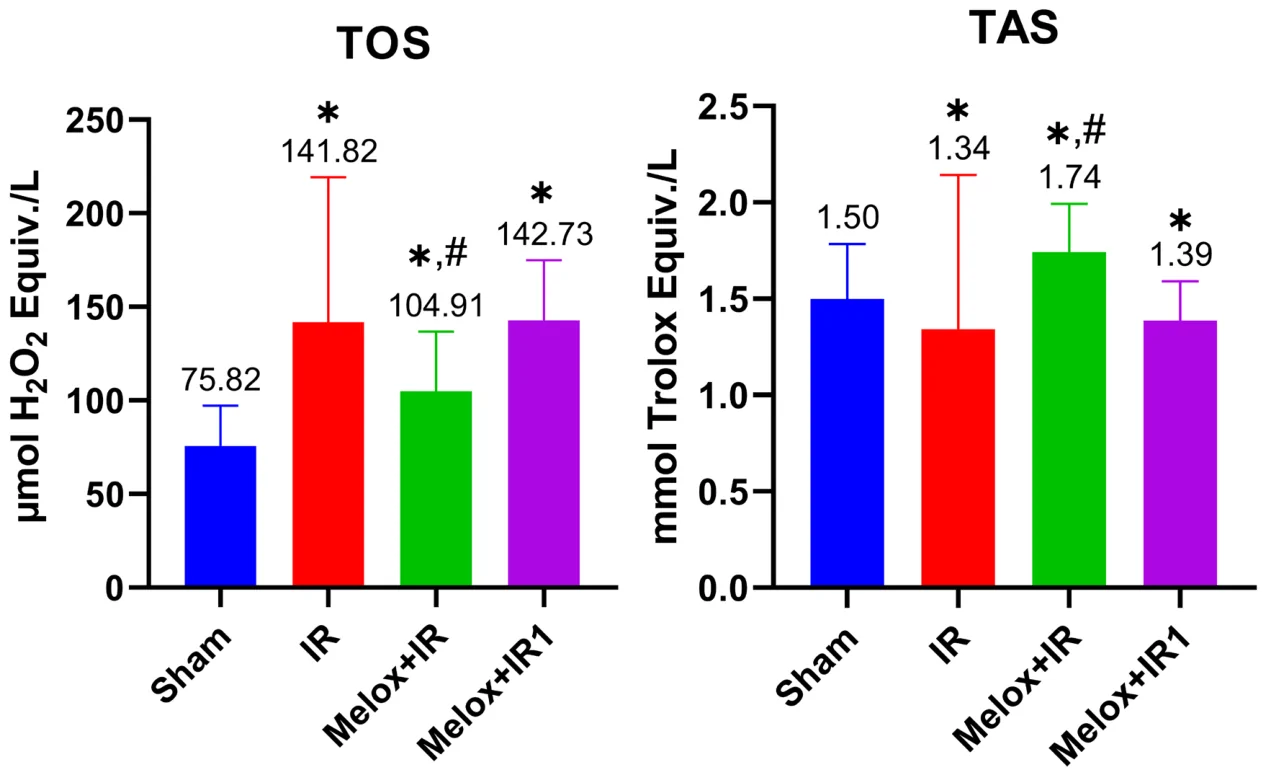
Blood serum TOS and TAS levels. Data are presented as mean ± standard deviation (SD) (*n = 6* per group). * *p* < 0.05 indicates significant differences compared to the Sham group; # *p* < 0.05 indicates significant differences compared to the IR group (One-way ANOVA followed by Tukey’s post hoc test).

**Figure 6:**
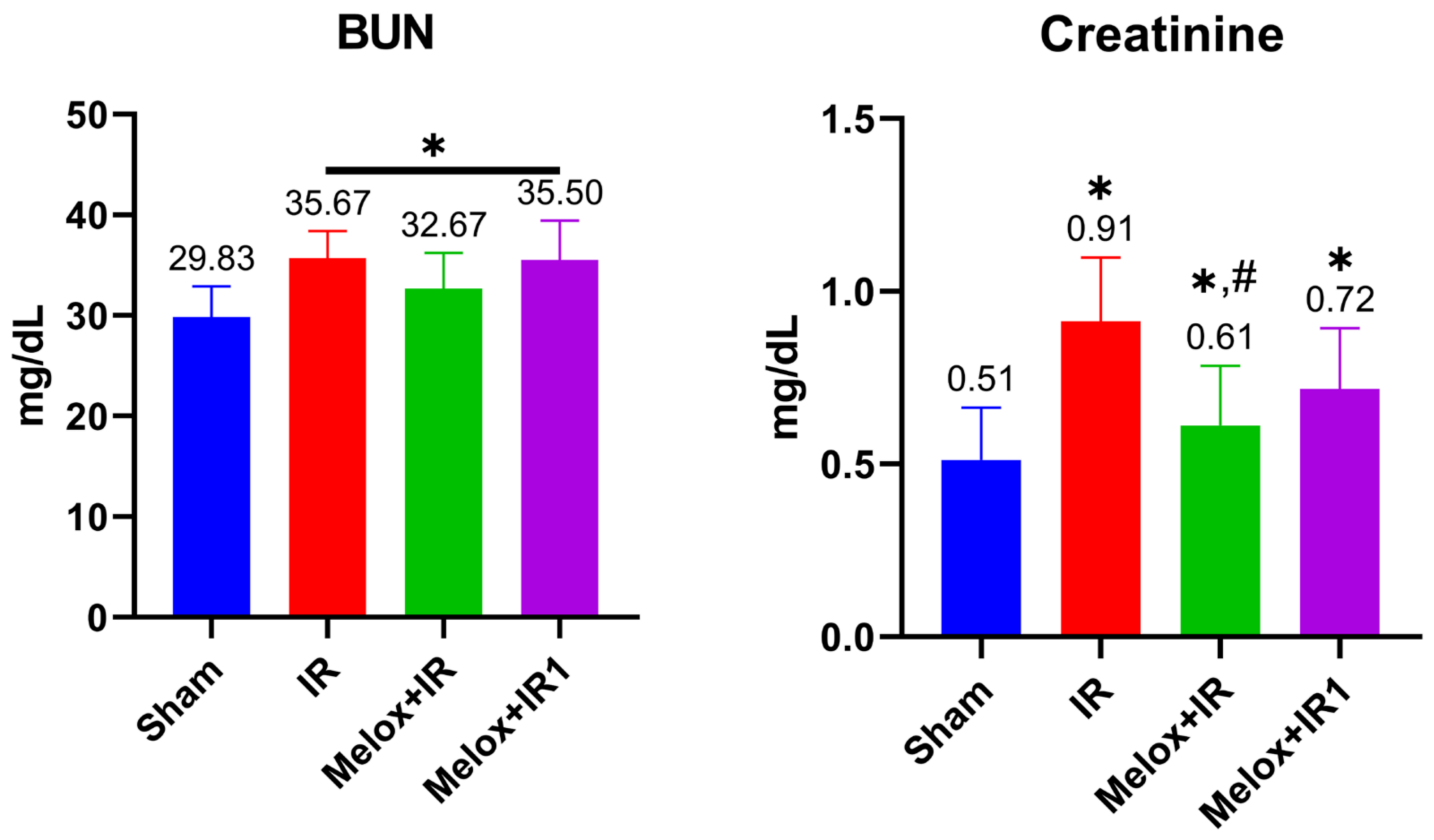
Blood serum BUN and Creatinine levels. Data are presented as mean ± standard deviation (SD) (*n = 6* per group). * *p* < 0.05 indicates significant differences compared to the Sham group; # *p* < 0.05 indicates significant differences compared to the IR group (One-way ANOVA followed by Tukey’s post hoc test).

Previous studies have demonstrated that tissue damage paradoxically initiates during the reperfusion phase of IR injury, accompanied by an increase in ROS derivatives [35,36]. Oxidative tissue damage triggered by the onset of reperfusion was confirmed in our study by high TOS and low TAS levels in the IR group. The evaluation of oxidative stress dynamics indicates that early meloxicam administration (Melox + IR group) effectively shifts the systemic redox balance in favor of TAS by significantly suppressing TOS levels and preserving antioxidant capacity. Conversely, this therapeutic modulation is not observed to the same extent when meloxicam treatment is delayed (Melox + IR1 group). In the delayed treatment cohort, TOS levels remain closely aligned with those of the untreated ischemia–reperfusion (IR) group, and the statistical robusticity of TAS alterations is markedly limited by relatively wide error bars (Figure 5). These findings collectively demonstrate that while early intervention with meloxicam successfully mitigates oxidative stress and drives the balance toward antioxidant dominance, delayed administration offers restricted efficacy in restructuring the tissue redox state. Regarding renal functional parameters, while both early and delayed meloxicam treatments significantly improved serum creatinine levels, a corresponding significant reduction was not observed in BUN levels within the same 120 min reperfusion period (Figure 6). Rather than attributing this discrepancy solely to the relatively short 120 min timeline, this outcome can be more consistently explained by the intrinsic physiological differences between these two biomarkers. Serum creatinine is a highly specific and direct surrogate marker of glomerular filtration rate (GFR) in acute settings. Conversely, BUN is a much more volatile biomarker that is heavily influenced by non-renal factors. Specifically, surgical stress-induced protein catabolism, hydration status, and the neurohumoral effects of anesthesia can transiently elevate or maintain high BUN levels, which may mask or lag behind the rapid recovery of GFR reflected by creatinine. Therefore, the stable BUN levels observed at 120 min of reperfusion likely reflect these extra-renal confounding variables rather than a lack of therapeutic efficacy. Conversely, the improvement observed in the meloxicam-treated groups compared to the IR group in levels of Cystatin C and KIM-1 which are early markers of functional loss resulting from renal tissue damage parallels the other data obtained from our study.

### 4.2. Evaluation of the Inflammatory Process

**Figure 7:**
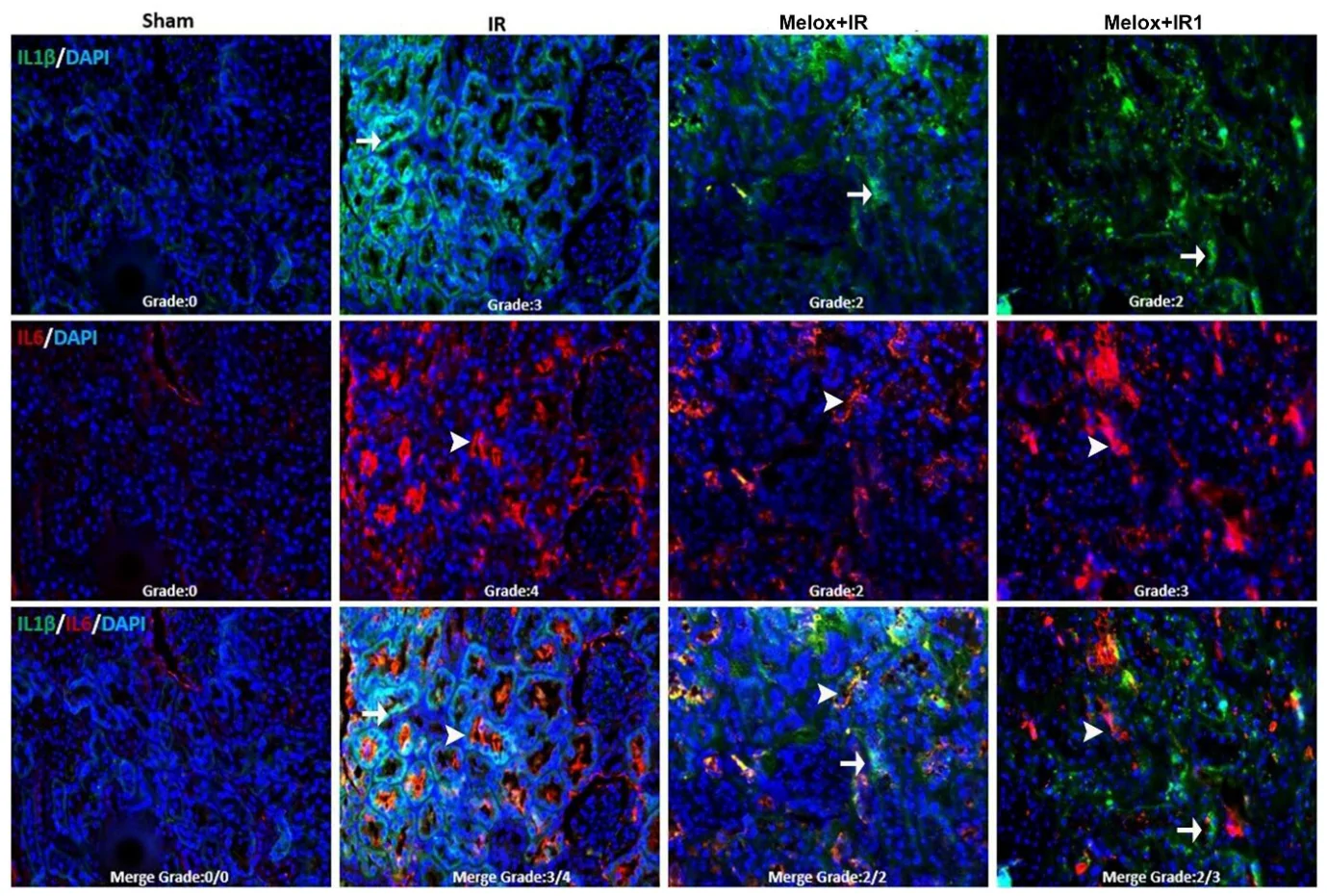
IL-1β positivity: negative in the Sham group, severe in the IR group, and moderate in the Melox + IR and Melox + IR1 groups. IL-6 positivity: negative in the Sham group, highly severe in the IR group, moderate in the Melox + IR group, and severe in the Melox + IR1 group. IL-1β positivity: FITC (→), IL-6 positivity: Alexa Fluor 594 (➤), DAPI: 4′,6-diamidino-2-phenylindole. Grade 0 (absent), Grade 1 (mild), Grade 2 (moderate), Grade 3 (severe), and Grade 4 (highly severe).

**Figure 8:**
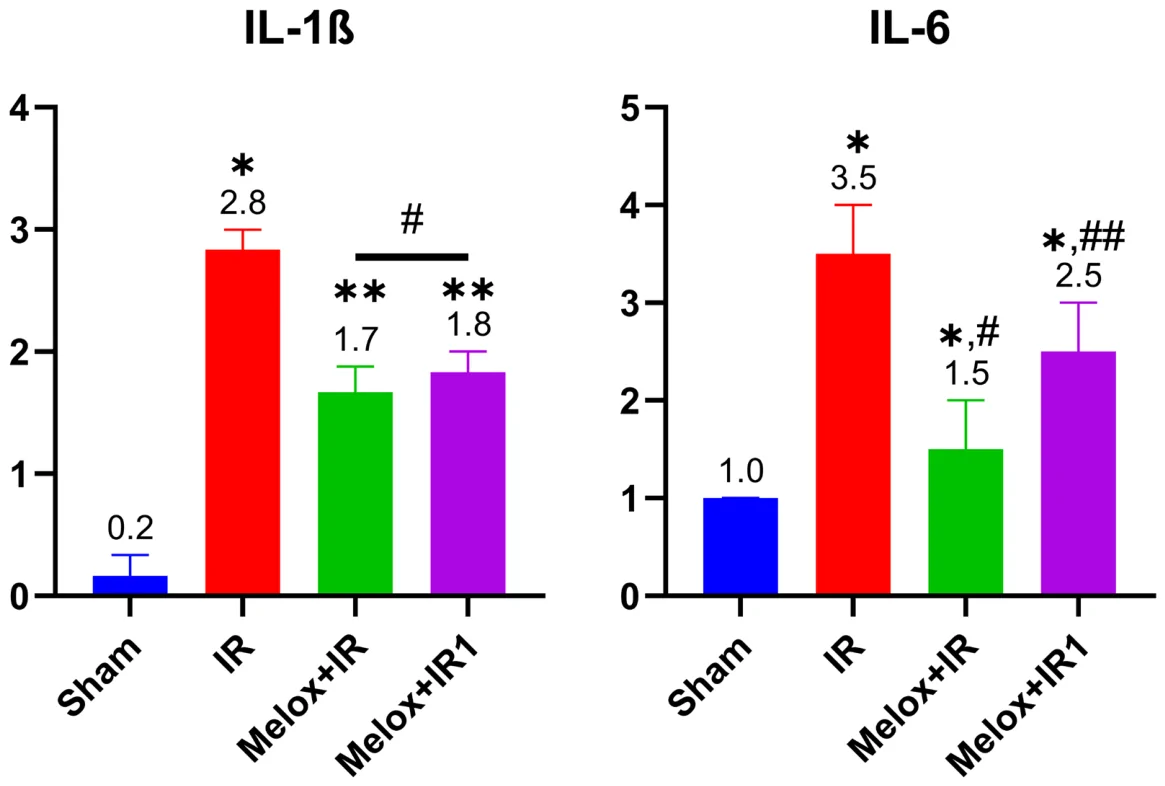
Effects of early and delayed meloxicam treatment on renal tissue IL-1β and IL-6 levels. Data are presented as median with interquartile range (IQR) with individual data points plotted (*n = 6* per group). * *p* < 0.05; ** *p* < 0.01 compared to the Sham group; # *p* < 0.05, ## *p* < 0.01 compared to the IR group (Kruskal–Wallis followed by Dunn’s post hoc test).

It is well established that interleukin IL-6 and IL-1β exert pro-inflammatory effects within inflammatory processes, whereas IL-10 exhibits anti-inflammatory properties [37,38,39]. The prominent increase in IL-6 and IL-1β in the inflammatory cascade within the IR group is viewed as a consequence of reperfusion-induced neutrophil infiltration. The capacity of meloxicam to suppress these proinflammatory cytokines while stabilizing tissue homeostasis-supporting IL-10 levels suggests that it facilitates the transition of inflammation from an active inflammatory state to a resolution phase. The observation that IL-6 levels in the Melox + IR group approached the Sham group levels indicates that meloxicam administration at the initial stage of reperfusion may be more effective in establishing anti-inflammatory efficacy (Figure 7 and Figure 8). Similarly, IL-10 levels rose in meloxicam-treated groups compared to the IR group. However, it was noted that the increase in anti-inflammatory IL-10 levels achieved when meloxicam was administered immediately at the onset of reperfusion could not be replicated when administered 1 h post reperfusion.

**Figure 9:**
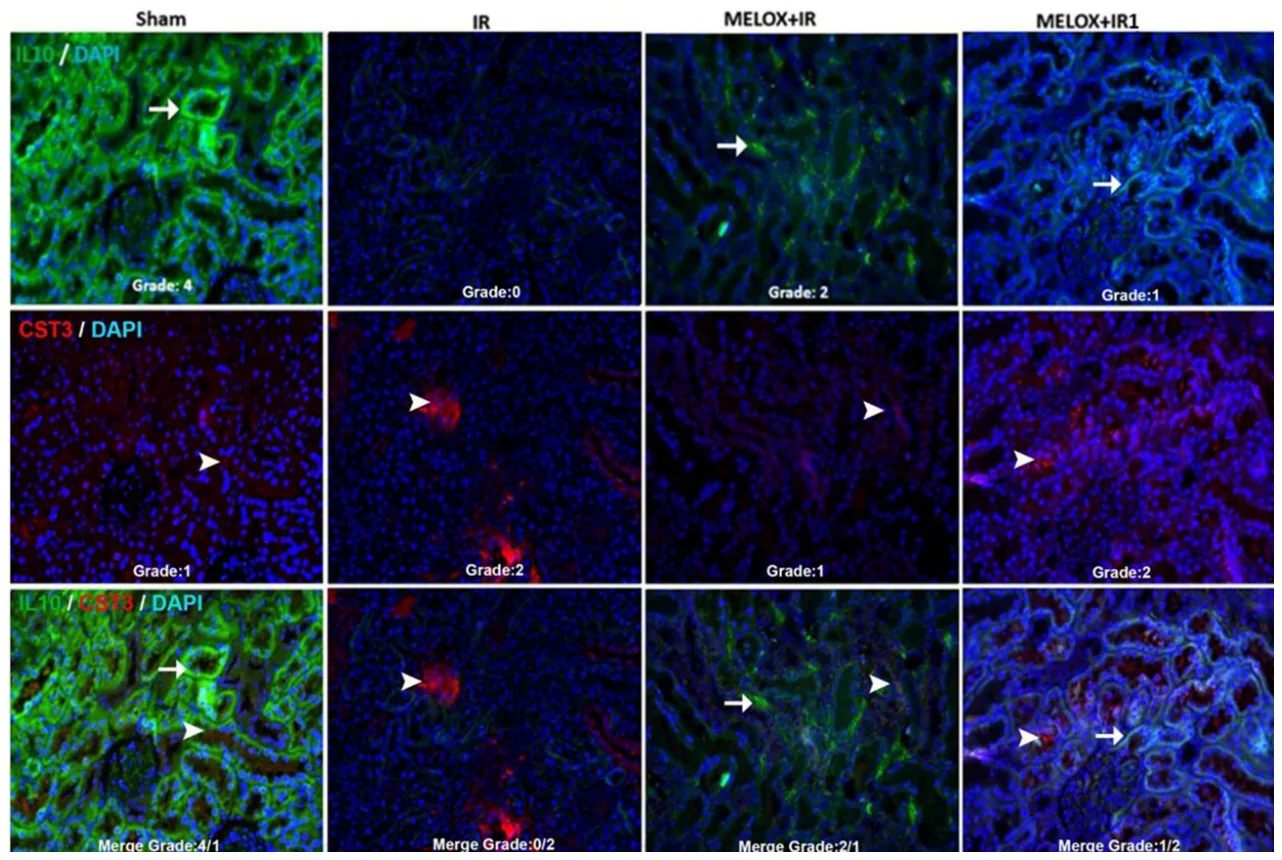
IL-10 positivity: highly severe in the Sham group, negative in the IR group, moderate in the Melox + IR group, and mild in the Melox + IR1 group. CST C (CST3) positivity: mild in the Sham group, moderate in the IR group, mild in the Melox + IR group, and moderate in the Melox + IR1 group. IL-10 positivity: FITC (→), CST C positivity: Alexa Fluor 594 (➤), DAPI: 4′,6-diamidino-2-phenylindole. Grade 0 (absent), Grade 1 (mild), Grade 2 (moderate), Grade 3 (severe), and Grade 4 (highly severe).

**Figure 10:**
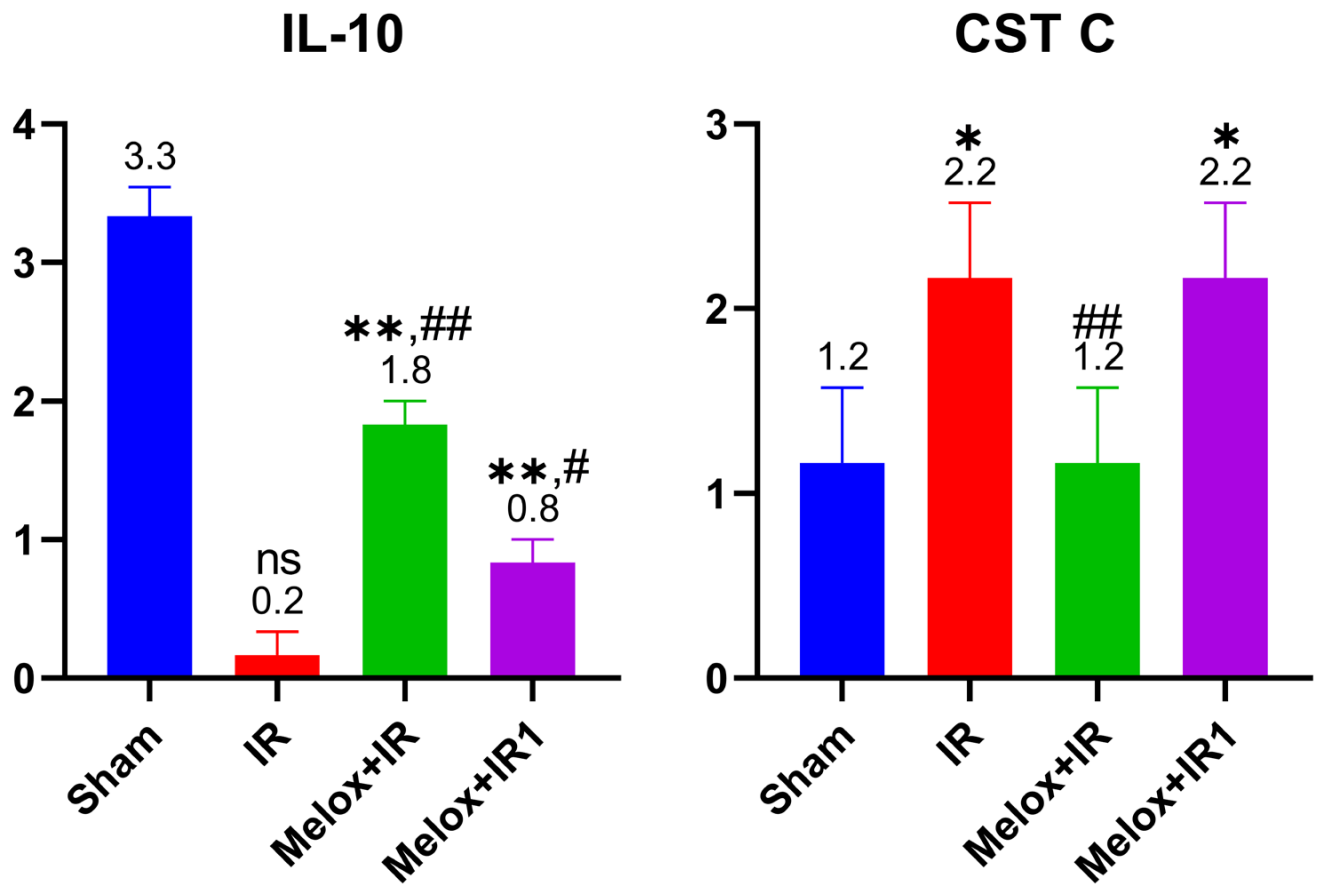
Effects meloxicam treatment on renal tissue IL-10 and CST C levels. Data are presented as median range with individual data points plotted (*n = 6* per group). * *p* < 0.05; ** *p* < 0.01 compared to the Sham group; # *p* < 0.05; ## *p* < 0.01 compared to the IR group (Kruskal–Wallis followed by Dunn’s post hoc test). ns, not significant.

The immunohistochemical evaluation revealed that IL-10 staining was highest in the Sham group and dropped to almost undetectable levels in the IR group (Figure 9 and Figure 10). Physiologically, IL-10 serves as a crucial baseline anti-inflammatory cytokine that limits the host immune response to prevent excessive tissue injury. The near total depletion of IL-10 expression in the IR group reflects the massive, unchecked pro-inflammatory cascade triggered by reperfusion injury, which overpowers and exhausts local anti-inflammatory defenses. Intriguingly, early meloxicam administration significantly preserved IL-10 immunoreactivity. Rather than acting as a direct stimulant for IL-10 production, meloxicam’s “protective” effect on IL-10 can be attributed to its selective COX-2 inhibition. By suppressing excessive prostaglandin E2 (PGE2) downstream signaling and mitigating the acute influx of pro-inflammatory cytokines like IL-1β and IL-6, meloxicam prevents the inflammatory overactivation that would otherwise exhaust endogenous anti-inflammatory reservoirs. Consequently, meloxicam preserves the baseline anti-inflammatory microenvironment, allowing IL-10 to maintain its protective signaling pathways and mitigate subsequent renal tubular apoptosis.

The anti-inflammatory efficacy of meloxicam observed in this study warrants a careful discussion regarding the well-known COX-2 paradox in renal physiology. Constitutively expressed and stress-induced COX-2-derived prostaglandins, particularly PGE2 and prostacyclin, are widely recognized to play a critical role in maintaining renal hemodynamics and vascular autoregulation under ischemic conditions [40]. Consequently, inhibiting COX-2 during renal stress raises theoretical concerns regarding compromised renal perfusion and exacerbated nephrotoxicity. However, the renoprotection demonstrated by meloxicam in our experimental model can be explained by two key factors. First, during the acute phase of ischemia–reperfusion injury, the uncontrolled and pathological up-regulation of COX-2 leads to an excessive production of pro-inflammatory mediators and subsequent neutrophil infiltration, which heavily outweighs its physiological vasodilator benefits [33]. Therefore, we suggest that selective COX-2 inhibition achieved by administering meloxicam at the onset of reperfusion helps suppress this devastating inflammatory cascade, thereby shielding the renal tissue from subsequent inflammatory injury. Second, the therapeutic benefits of meloxicam likely extend beyond simple COX inhibition. Based on the findings of the present study, meloxicam shows potential associations with pathways beyond standard COX-2 inhibition, including reductions in markers of reactive oxygen species and the modulation of intracellular cascades such as the MAPK/ERK pathway. This associated multi-pathway profile suggests that meloxicam may support renal cell survival across certain regulated cell death pathways potentially independently of prostaglandin dependent mechanisms, thereby offering new insights into tissue preservation dynamics.

### 4.3. Suppression of Necroptosis: Discussion of the RIPK3 and MLKL Axis

**Figure 11:**
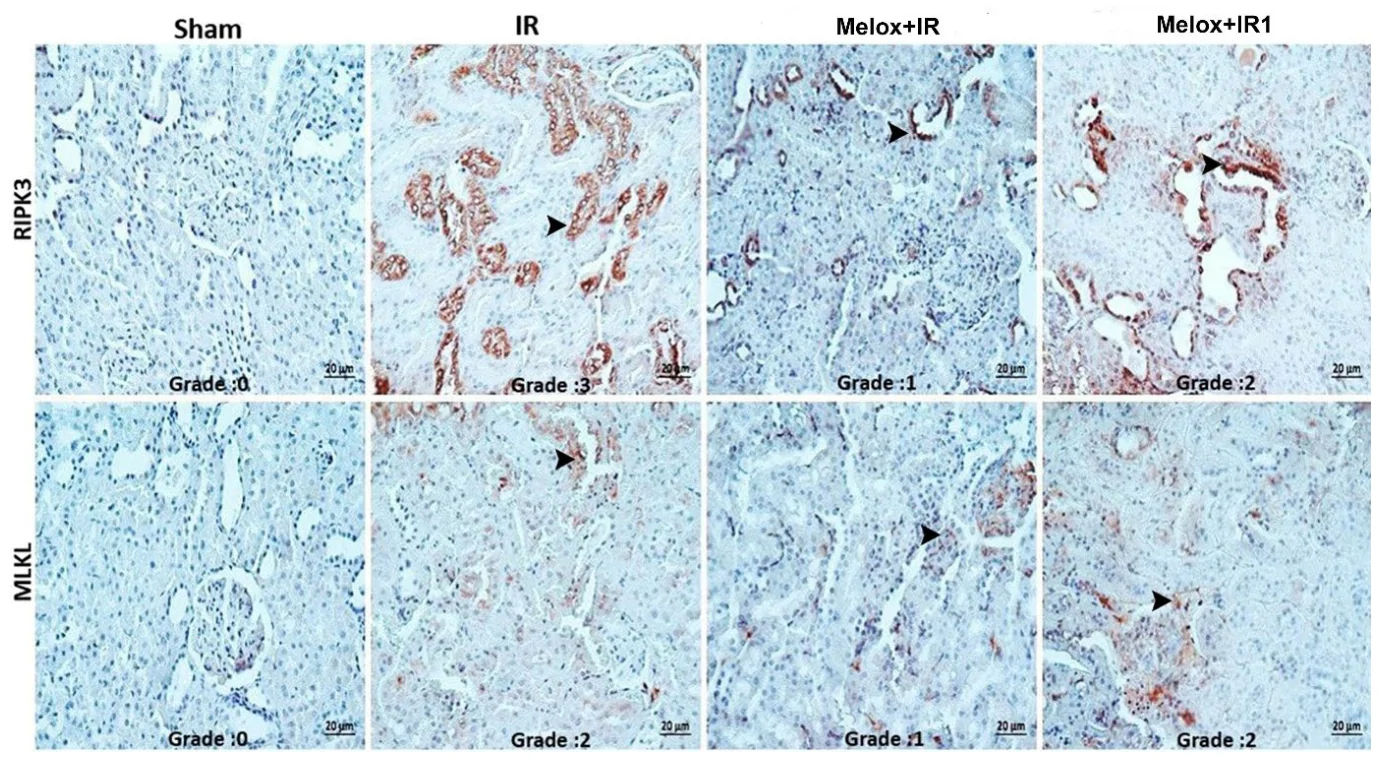
RIPK3 positivity: negative in the Sham group, severe in the IR group, mild in the Melox + IR group, and moderate in the Melox + IR1 group. MLKL positivity: negative in the Sham group, moderate in the IR group, mild in the Melox + IR group, and moderate in the Melox + IR1 group (➤). Grade 0 (absent), Grade 1 (mild), Grade 2 (moderate), and Grade 3 (severe).

**Figure 12:**
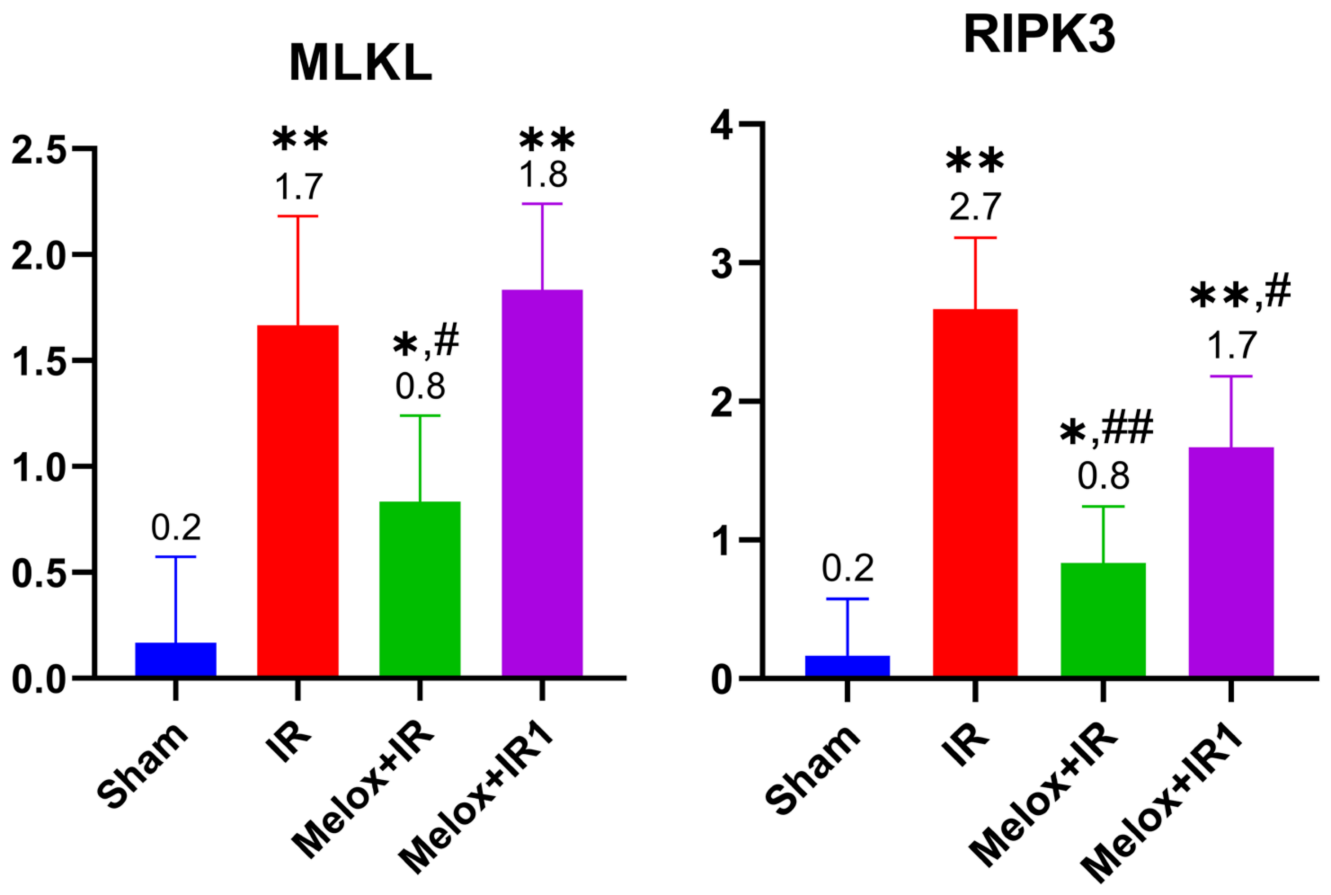
MLKL and RIPK3 levels on renal tissue. Data are presented as median range with individual data points plotted (*n = 6* per group). * *p* < 0.05; ** *p* < 0.01 compared to the Sham group; # *p* < 0.05; ## *p* < 0.01 compared to the IR group (Kruskal–Wallis followed by Dunn’s post hoc test).

In contrast to accidental necrosis, necroptosis is defined as a controlled form of cell death and is thought to play a pivotal role in the pathogenesis of I/R injury [41,42,43]. The downregulation of RIPK3 and MLKL expressions detected via immunofluorescence in the meloxicam treatment groups serves as concrete molecular evidence that meloxicam preserves cell membrane integrity [41,43,44]. Pores formed by the phosphorylation and subsequent cell-membrane translocation of MLKL cause cytoplasmic content leakage and trigger the necroinflammation cycle [45,46]. Meloxicam’s disruption of this axis underscores its role as a critical barrier for renal tubular epithelial cell survival. The greater decrease in necroptosis markers in the Melox + IR group compared to the delayed Melox + IR1 group emphasizes that early intervention with meloxicam shields cells more robustly against necroptosis (Figure 11 and Figure 12).

### 4.4. Significance and Discussion of MAPK/ERK1/2 Signaling and Regulated Cell Death Pathways

**Figure 13:**
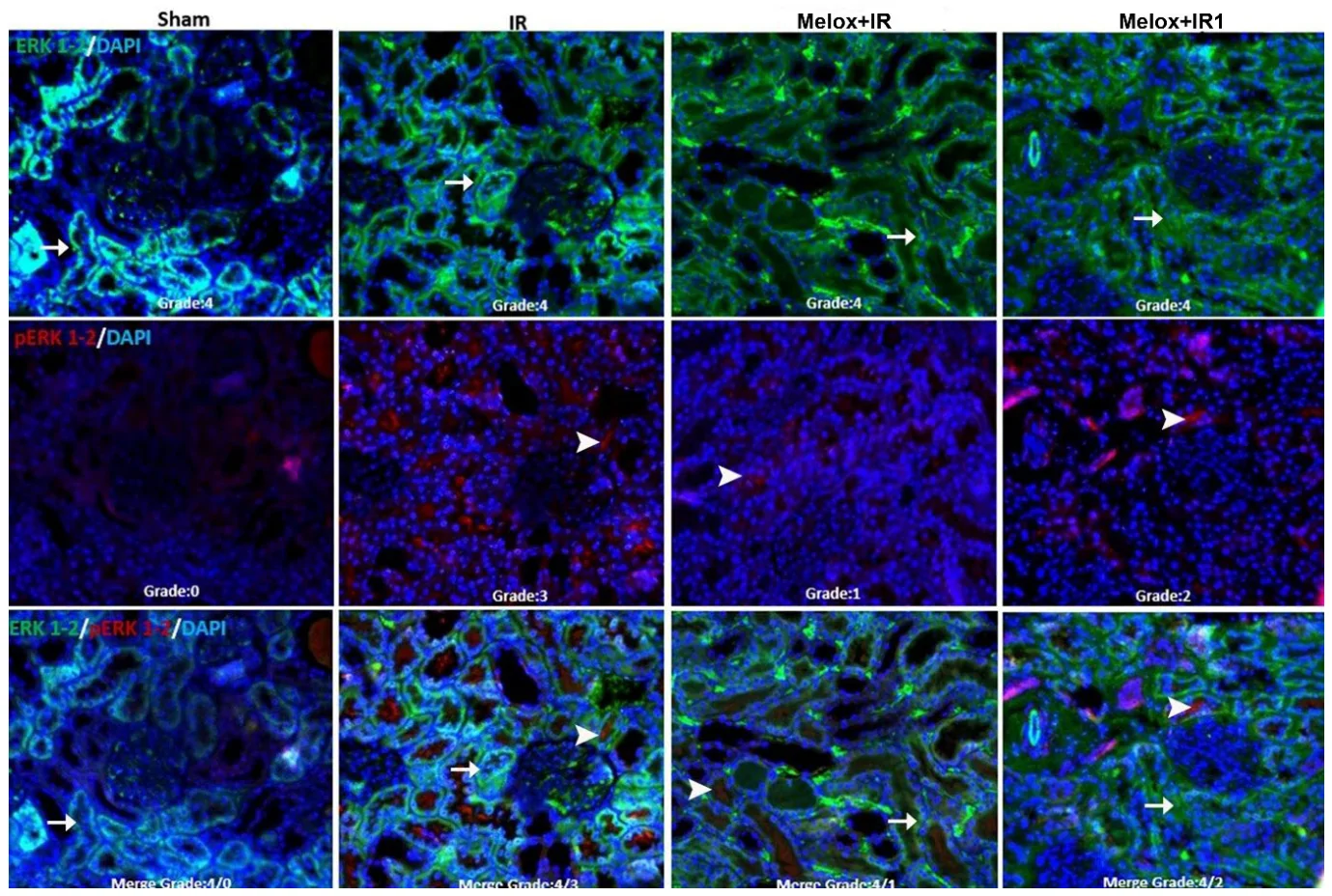
ERK1/2 positivity: highly severe positivity across Sham, IR, Melox + IR, and Melox + IR1 groups. pERK1/2 positivity: negative in the Sham group, severe in the IR group, mild in the Melox + IR group, and moderate in the Melox + IR1 group. ERK1/2 positivity: FITC (→), pERK1/2 positivity: Alexa Fluor 594 (➤), DAPI: 4′,6-diamidino-2-phenylindole. Grade 0 (absent), Grade 1 (mild), Grade 2 (moderate), Grade 3 (severe), and Grade 4 (highly severe).

**Figure 14:**
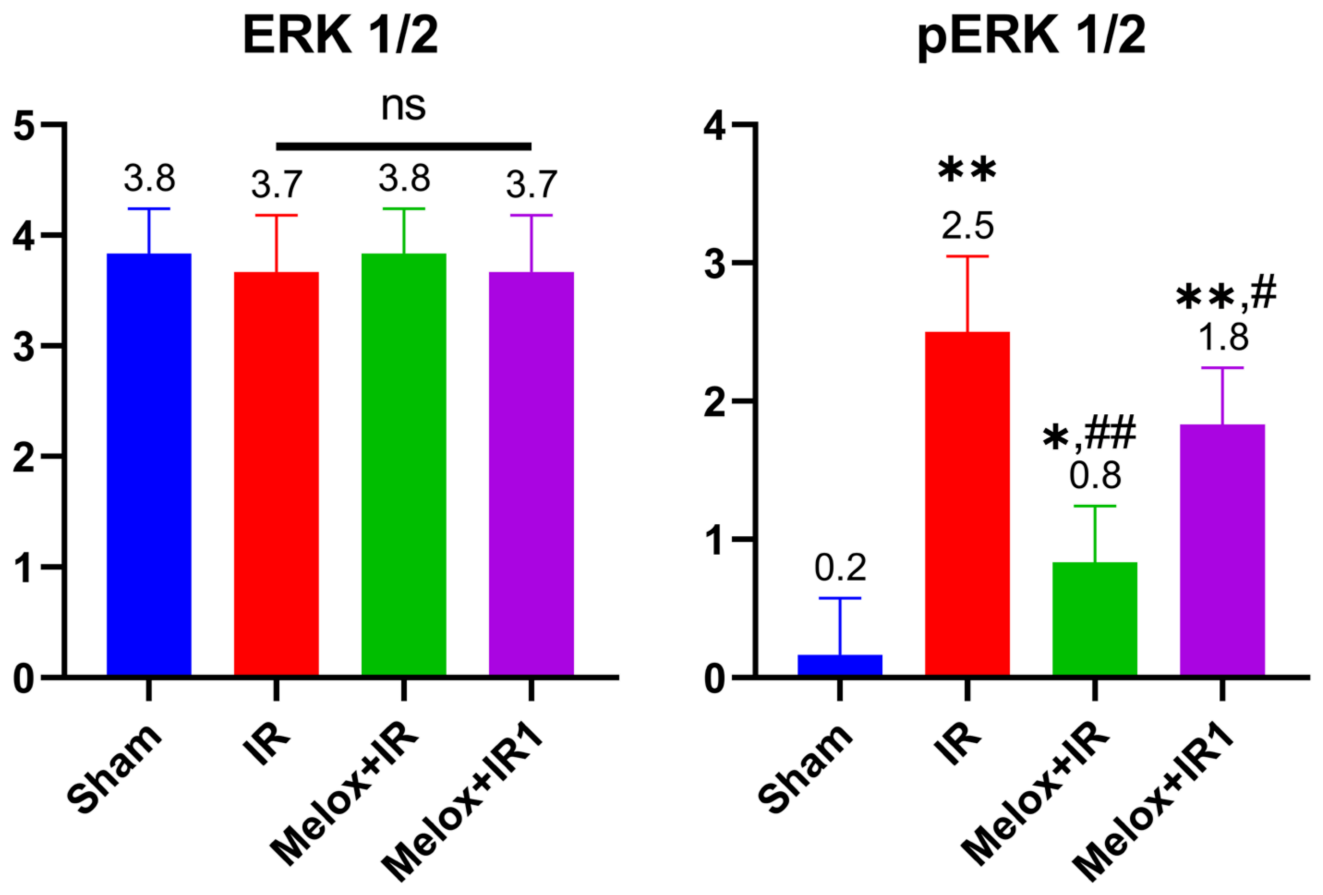
ERK1/2 and pERK1/2 levels on renal tissue. Data are presented as median range with individual data points plotted (*n = 6* per group). * *p* < 0.05; ** *p* < 0.01 compared to the Sham group; # *p* < 0.05; ## *p* < 0.01 compared to the IR group (Kruskal–Wallis followed by Dunn’s post hoc test). ns, not significant.

Ferroptosis is a recently discovered form of regulated cell death characterized by iron-dependent lipid peroxidation [20,47]. The phosphorylation and subsequent activation of ERK1/2 into pERK1/2 is a key signaling node within the MAPK cascade, and literature suggests it may also play a regulatory role in transmitting downstream death signals during cell injury [48,49]. In the present study, meloxicam administration significantly limited pERK1/2 activation in parallel with a reduction in tissue oxidative stress (Figure 13 and Figure 14). While these findings demonstrate that meloxicam modulates the MAPK/ERK pathway, they also suggest a potential protective effect against regulated cell death mechanisms such as ferroptosis, which are closely linked to oxidative damage. However, because direct and canonical markers of ferroptosis or tissue iron accumulation were not evaluated in our study, a definitive conclusion regarding meloxicam’s direct modulation of ferroptosis cannot be made. Nevertheless, the more profound suppression of pERK1/2 in the early treatment group relative to the delayed group highlights the time dependent efficacy of meloxicam in preserving cellular signaling homeostasis. This modulation of the MAPK pathway introduces a valuable perspective to the known pharmacological profile of meloxicam in renal injury.

### 4.5. Discussion of Functional Recovery and Apoptosis

**Figure 15:**
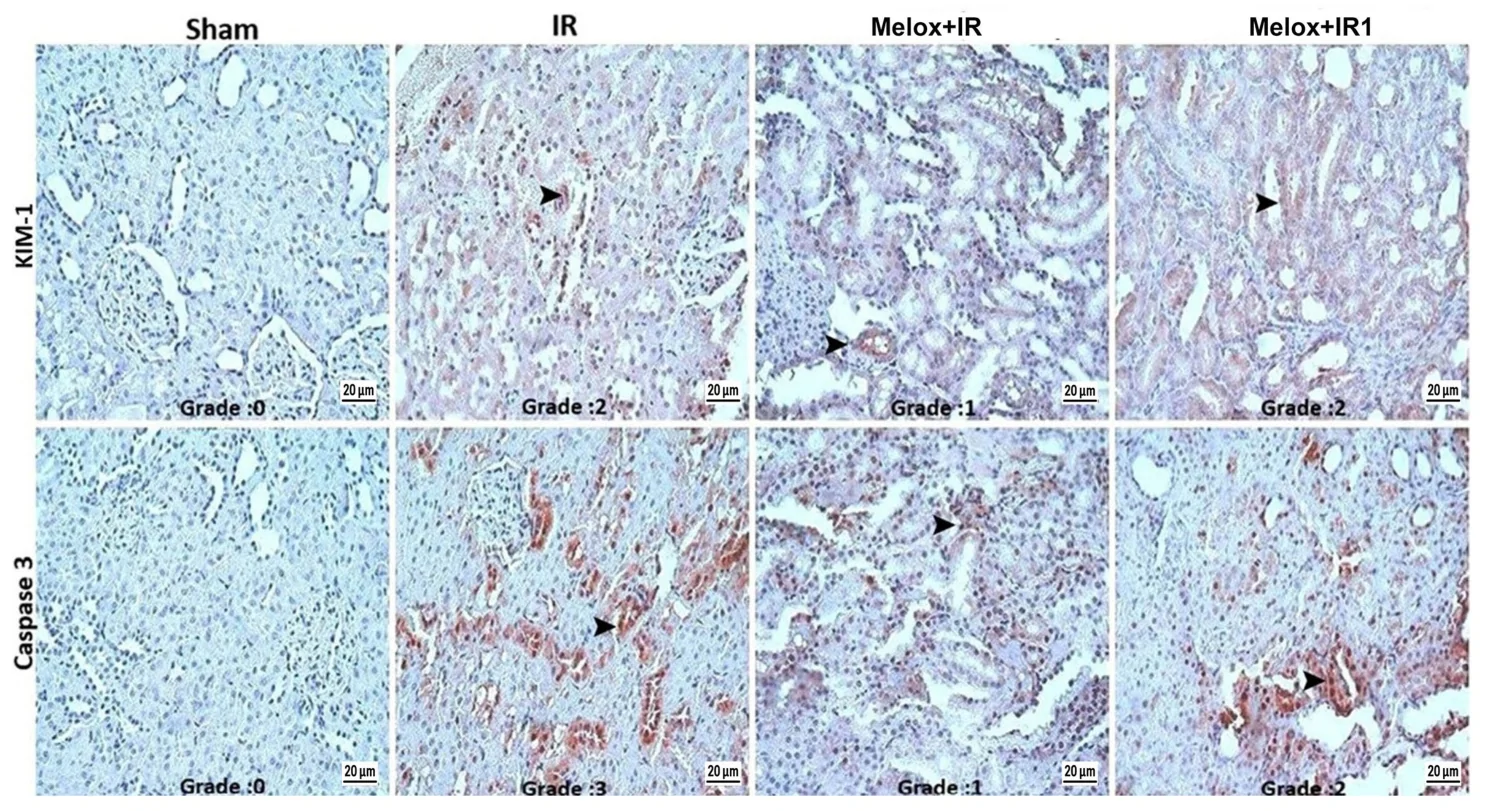
KIM-1 positivity: negative in the Sham group, moderate in the IR group, mild in the Melox + IR group, and moderate in the Melox + IR1 group. Caspase 3 positivity: negative in the Sham group, severe in the IR group, mild in the Melox + IR group, and moderate in the Melox + IR1 group (➤). Grade 0 (absent), Grade 1 (mild), Grade 2 (moderate), and Grade 3 (severe).

The restoration of Cystatin C and KIM-1 levels, one of our most clinically vital findings, can be interpreted as the functional translation of molecular protection [50,51]. While KIM-1 serves as an early indicator of tubular epithelial injury, Cystatin C mirrors alterations in glomerular filtration rate with higher sensitivity than Creatinine [52,53,54]. In light of our data, meloxicam’s ability to bring these markers closer to the Sham group values demonstrates that functional injury induced by 40 min of ischemia can be significantly reversed when a 1 mg/kg dose is applied at the onset of the reperfusion phase (Figure 9 and Figure 15).

**Figure 16:**
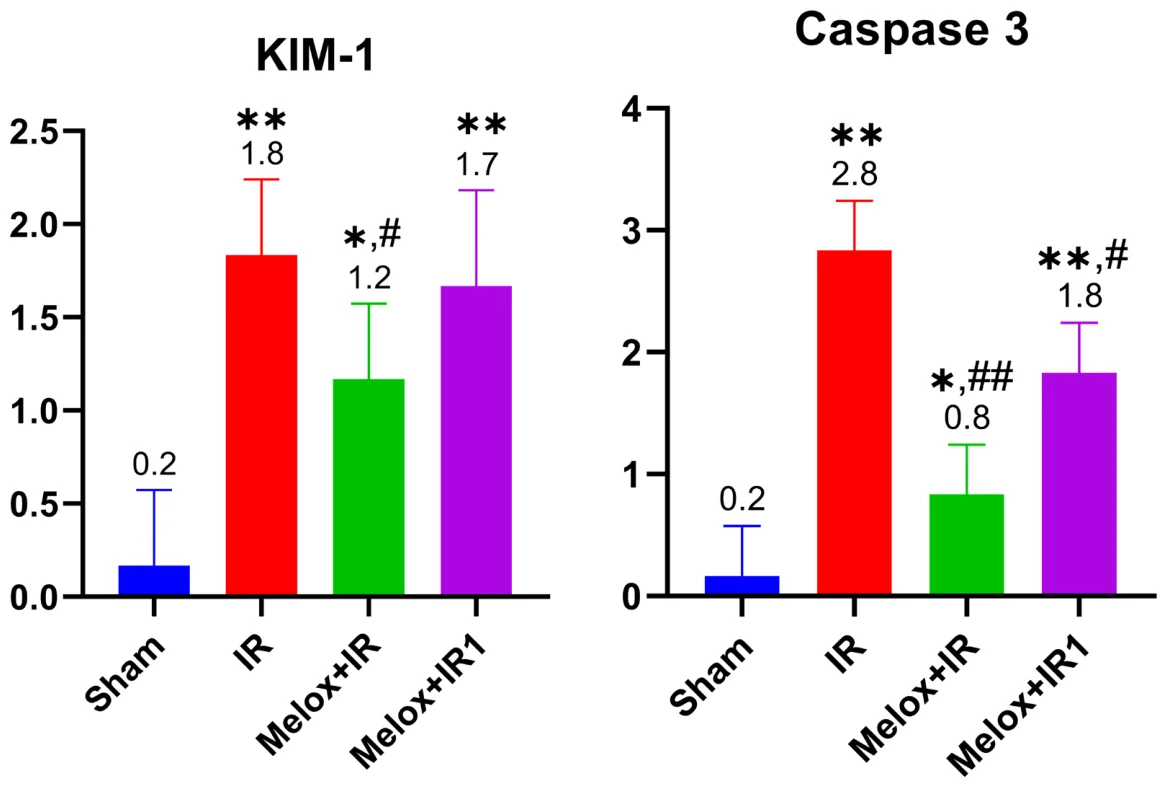
KIM-1 and Caspase 3 statistical analysis. Data are presented as median range with individual data points plotted (*n = 6* per group). * *p* < 0.05; ** *p* < 0.01 compared to the Sham group; # *p* < 0.05; ## *p* < 0.01 compared to the IR group (Kruskal–Wallis followed by Dunn’s post hoc test).

Apoptosis, known as programmed cell death, is executed via Caspase-3, an executive caspase activated by intrinsic or extrinsic cell death signals [55,56]. While apoptotic pathways naturally initiate during cellular senescence, they are also reported to emerge due to ischemic conditions, pathogens, or irreparable damage caused by toxic chemical compounds [57,58]. Regardless of the trigger, apoptosis is characterized by a cascade of caspase enzymes culminating in DNA fragmentation by Caspase 3 [59,60]. In this study, renal tissue apoptosis levels following 40 min of ischemia and reperfusion-phase meloxicam treatment were semi-quantitatively analyzed. Meloxicam administration during the reperfusion phase significantly suppressed apoptosis in kidney tissues compared to the IR group. Additionally, administering meloxicam at the immediate onset of the reperfusion phase protected cells from apoptosis more effectively than delaying administration by 1 h (Figure 15 and Figure 16). These data obtained from our study are consistent with earlier reports demonstrating the anti-apoptotic effects of meloxicam [33].

When comparing our findings with the existing literature, the protective potential of COX-2 inhibitors against renal I/R injury shows both notable parallels and key differences. Previous studies evaluating selective COX-2 inhibitors, such as celecoxib or meloxicam, have consistently reported a reduction in overall apoptotic markers and inflammatory cytokines in various ischemic models [61,62]. Similarly, our study demonstrated a down regulation of Caspase 3 and an attenuation of tissue injury scores. However, distinct differences emerge regarding the timing of administration and the specific signaling pathways involved. While earlier research primarily focuses on pre-ischemic treatment protocols, our findings highlight that post ischemic intervention specifically at the 61st minute of reperfusion yields a unique regulatory trend. Furthermore, while conventional literature often attributes the protective effects of COX-2 inhibitors solely to prostaglandin suppression [62], our data suggest an associated modulation of downstream cascades such as the RIPK3/MLKL necroptotic pathway and p-ERK1/2 signaling. These discrepancies suggest that the therapeutic window and mechanistic pathways of meloxicam in renal I/R are highly sensitive to intervention timing, pointing toward non canonical, COX-independent dynamics that warrant deeper investigation.

## 5. Conclusions

This study comprehensively reveals the therapeutic potential of meloxicam in the acute pathogenesis of renal I/R injury, extending beyond its traditional anti-inflammatory actions to embrace modern cell death mechanisms. The gathered data indicate that 1 mg/kg meloxicam administered at the onset of reperfusion suppresses oxidative stress developing in kidney tissue and reinforces antioxidant capacity, thereby sustaining cellular homeostasis. Conclusively, our findings demonstrate that meloxicam inhibits necroptosis via the RIPK3/MLKL axis and apoptosis by reducing Caspase 3 levels, while also modulating the pERK1/2 signaling pathway, which represents a potential link to other regulated cell death processes such as ferroptosis. Our findings align with prior research evaluating the effectiveness of COX-2 inhibitors against ischemic injury [62].

Across nearly all biochemical and molecular parameters analyzed, administering meloxicam at the immediate onset of the reperfusion phase appeared to be more effective in terms of protective properties compared to administration delayed by 1 h. This suggests that meloxicam may serve as a pleiotropic agent capable of modulating specific cell death pathways rather than acting solely as a COX-2 inhibitor. Furthermore, the significant improvement in KIM-1 and Cystatin C levels, early and sensitive indicators of renal injury, points to the success of this molecular preservation in stabilizing renal functional capacity.

In conclusion, our study demonstrates that meloxicam administered after 40 min of ischemia attenuates the severity of subsequent acute kidney injury by shielding renal epithelial cells from apoptotic and necroptotic cell death, while also modulating key pERK1/2 mediated signaling pathways. Rather than representing an immediate clinical solution, these preliminary rodent findings suggest that early selective COX-2 modulation warrants further investigation as a potential supportive pharmacological candidate in renal ischemic stress. Future long term studies utilizing broad dose–response analyses, extended reperfusion windows, and functional survival assessments will be essential to clarify whether these molecular benefits can translate into safe and effective clinical applications during high-risk surgical procedures or renal transplantation.

Although this study provides comprehensive histopathological and immunohistochemical insights into the renal protective effects of early and delayed meloxicam, certain limitations should be acknowledged. First, the evaluation of key apoptosis, necroptosis, and MAPK signaling pathways relies on semi-quantitative scoring and fluorescence intensity, which lack validation by definitive quantitative molecular methods such as Western blotting or qPCR. Second, rather than longitudinal urinalysis, the clinical gold standard for acute kidney injury kinetics, KIM-1 expression was evaluated solely through localized tissue immunofluorescence staining. Third, the study did not evaluate a range of drug doses or alternative administration schedules, limiting our ability to establish an optimized dose–response profile or a precise therapeutic window. Finally, we only assessed acute renal outcomes within a 120 min reperfusion window; therefore, the long-term functional and structural recovery or potential chronic sequelae of meloxicam treatment remain unexamined. Future studies incorporating quantitative molecular techniques, urinary kinetic profiles, dose–response ranges, and long-term follow-up are warranted to address these aspects.

## Figures and Tables

**Table 1:** Antibodies used for immunohistochemistry (IHC) and immunofluorescence (IF) analyses.

Antibody	Manufacturer	Host	Catalog No
KIM-1	Bioss (Woburn, MA, USA)	Rabbit	BS-2713R
Caspase 3	Thermofisher (Waltham, MA, USA)	Rabbit	PA5-114687
RIPK3	BT-Lab (Shanghai, China)	Rabbit	BT-AP12295
MLKL	BT-Lab (Shanghai, China)	Rabbit	BT-AP11379
Monoclonal IL1β	Santa cruz (Santa Cruz, CA, USA)	Mouse	SC-52012
Polyclonal IL6	Affbiotech (Cincinnati, OH, USA)	Rabbit	AF6087
Monoclonal IL10	Santa cruz (Santa Cruz, CA, USA)	Mouse	SC-8438
Polyclonal CST3	Bioss (Woburn, MA, USA)	Rabbit	BS-3595R
Monoclonal ERK1/2	Santa cruz (Santa Cruz, CA, USA)	Mouse	SC-514302
Polyclonal pERK1/2	Affbiotech (Cincinnati, OH, USA)	Rabbit	AF1015

## Data Availability

The data presented in this study are available on request from the corresponding author due to ethical restrictions.

## Abbreviations

AKI	Acute Kidney Injury
BUN	Blood Urea Nitrogen
COX-2	Cyclooxygenase-2
DAB	3,3′-Diaminobenzidine
ERK1/2	Extracellular signal-regulated kinase 1/2
pERK1/2	Phosphorylated Extracellular signal-regulated kinase 1/2
GFR	Glomerular Filtration Rate
I/R	Ischemia and Reperfusion
KIM-1	Kidney Injury Molecule-1
MAPK	Mitogen Activated Protein Kinase
MLKL	Mixed Lineage Kinase Domain Like Pseudokinase
NSAID	Non-Steroidal Anti-Inflammatory Drug
PGE2	Prostaglandin E2
RIPK-3	Receptor-interacting protein kinase-3
RCD	Regulated cell death
ROS	Reactive Oxygen Species
TAS	Total Anti-oxidative Status
TOS	Total Oxidative Status

## References

[1] Zhao H., Alam A., Soo A.P., George A.J., Ma D. (2018). Ischemia-reperfusion injury reduces long term renal graft survival: Mechanism and beyond. eBioMedicine.

[2] Mohsen I.H., Maaroof R.J., HarjanMohsen A. (2023). Renal failure, types, causes and etiology: A review article. Int. J. Med. Sci. Clin. Res. Stud..

[3] Nieuwenhuijs-Moeke G.J., Pischke S.E., Berger S.P., Sanders J.S.F., Pol R.A., Struys M.M., Ploeg R.J., Leuvenink H.G. (2020). Ischemia and reperfusion injury in kidney transplantation: Relevant mechanisms in injury and repair. J. Clin. Med..

[4] Ma H., Guo X., Cui S., Wu Y., Zhang Y., Shen X., Xie C., Li J. (2022). Dephosphorylation of AMP-activated protein kinase exacerbates ischemia/reperfusion-induced acute kidney injury via mitochondrial dysfunction. Kidney Int..

[5] Basile D.P., Anderson M.D., Sutton T.A. (2012). Pathophysiology of acute kidney injury. Compr. Physiol..

[6] Faucher Q., Alarcan H., Marquet P., Barin-Le Guellec C. (2020). Effects of ischemia-reperfusion on tubular cell membrane transporters and consequences in kidney transplantation. J. Clin. Med..

[7] Feng Q., Yu X., Qiao Y., Pan S., Wang R., Zheng B., Wang H., Ren K.-D., Liu H., Yang Y. (2022). Ferroptosis and acute kidney injury (AKI): Molecular mechanisms and therapeutic potentials. Front. Pharmacol..

[8] Wang X., Tan X., Zhang J., Wu J., Shi H. (2023). The emerging roles of MAPK-AMPK in ferroptosis regulatory network. Cell Commun. Signal..

[9] Guerrero-Mauvecin J., Villar-Gómez N., Rayego-Mateos S., Ramos A.M., Ruiz-Ortega M., Ortiz A., Sanz A.B. (2023). Regulated necrosis role in inflammation and repair in acute kidney injury. Front. Immunol..

[10] Zhou Y., Yang Y., Wang B., Chen W., Wei Y., Wu R., Meng L., Lyu L. (2024). Discovery of ferroptosis-related genes in renal ischemia reperfusion and evaluate the potential impact on kidney transplantation. Front. Immunol..

[11] Liang Y., Liu Z., Qu L., Wang Y., Zhou Y., Liang L., Guo Y., Tang L. (2022). Inhibition of the IRE1/JNK pathway in renal tubular epithelial cells attenuates ferroptosis in acute kidney injury. Front. Pharmacol..

[12] Kim T.M., Lee K.W., Kim H.D., Hong S.O., Cho H.J., Yang J.H., Kim S.J., Park J.B. (2023). Evaluation of Selected Markers in Kidneys of Cynomolgus Monkey (*Macaca fascicularis*) with Induced Diabetes during Renal Ischemia-reperfusion Injury. Comp. Med..

[13] Chen C., Zheng M., Hou H., Fang S., Chen L., Yang J., Yao W., Zhang Q., Hei Z. (2022). Cellular senescence in ischemia/reperfusion injury. Cell Death Discov..

[14] Alsadder L., Hamadah A. (2025). Cardiac Ischaemia–Reperfusion Injury: Pathophysiology, Therapeutic Targets and Future Interventions. Biomedicines.

[15] Polichnowski A.J., Griffin K.A., Licea-Vargas H., Lan R., Picken M.M., Long J., Williamson G.A., Rosenberger C., Mathia S., Venkatachalam M.A. (2020). Pathophysiology of unilateral ischemia-reperfusion injury: Importance of renal counterbalance and implications for the AKI-CKD transition. Am. J. Physiol.-Ren. Physiol..

[16] Singh M.K., Yun H.R., Ranbhise J.S., Han S., Kim S.S., Kang I. (2026). Hypoxia, ROS, and HIF Signaling in I/R Injury: Implications and Future Prospects. Antioxidants.

[17] Peng P., Zou J., Zhong B., Zhang G., Zou X., Xie T. (2023). Protective effects and mechanisms of flavonoids in renal ischemia-reperfusion injury. Pharmacology.

[18] Liang Y., Qu L., Liu Z., Liang L., Wang Y., Quan S., Wang Y., Tang L. (2022). The IRE1/JNK signaling pathway regulates inflammation cytokines and production of glomerular extracellular matrix in the acute kidney injury to chronic kidney disease transition. Mol. Biol. Rep..

[19] Guo M., Chen Q., Huang Y., Wu Q., Zeng Y., Tan X., Teng F., Ma X., Pu Y., Huang W. (2023). High Glucose-Induced Kidney Injury via Activation of Necroptosis in Diabetic Kidney Disease. Oxid. Med. Cell. Longev..

[20] Thapa K., Singh T.G., Kaur A. (2022). Targeting ferroptosis in ischemia/reperfusion renal injury. Naunyn-Schmiedeberg’s Arch. Pharmacol..

[21] Wang J., Ma R., Wang Y., Zhang S., Wang J., Zheng J., Xue W., Ding X. (2023). RhMYDGF alleviates I/R-induced kidney injury by inhibiting inflammation and apoptosis via the Akt pathway. Transplantation.

[22] Samra M.M., Sadia A., Azam M., Imran M., Ahmad I., Basra M.A.R. (2022). Synthesis, spectroscopic and biological investigation of a new Ca (II) complex of meloxicam as potential COX-2 inhibitor. Arab. J. Sci. Eng..

[23] Schäufele T.J., Kolbinger A., Friedel J., Gurke R., Geisslinger G., Weigert A., Pierre S., Scholich K. (2024). Meloxicam treatment disrupts the regional structure of innate inflammation sites by targeting the pro-inflammatory effects of prostanoids. Br. J. Pharmacol..

[24] Zhang B., Han Y., Cheng M., Yan L., Gao K., Zhou D., Wang A., Lin P., Jin Y. (2025). Metabolomic effects of intrauterine meloxicam perfusion on histotroph in dairy heifers during diestrus. Front. Vet. Sci..

[25] Haffar A., Fillingham Y.A., Breckenridge L., Gursay D.A., Lonner J.H. (2022). Meloxicam versus celecoxib for postoperative analgesia after total knee arthroplasty: Safety, efficacy and cost. JAAOS Glob. Res. Rev..

[26] Ugidos I.F., González-Rodríguez P., Santos-Galdiano M., Font-Belmonte E., Anuncibay-Soto B., Pérez-Rodríguez D., Gonzalo-Orden J.M., Fernández-López A. (2023). Neuroprotective effects of meloxicam on transient brain ischemia in rats: The two faces of anti-inflammatory treatments. Neural Regen. Res..

[27] Cui L., Guo J., Wang Z., Zhang J., Li W., Dong J., Liu K., Guo L., Li J., Wang H. (2023). Meloxicam inhibited oxidative stress and inflammatory response of LPS-stimulated bovine endometrial epithelial cells through Nrf2 and NF-κB pathways. Int. Immunopharmacol..

[28] Karkoszka-Stanowska M., Rzepka Z., Wrześniok D. (2025). The Evaluation of Potential Anticancer Activity of Meloxicam—In Vitro Study on Amelanotic and Melanotic Melanoma. Int. J. Mol. Sci..

[29] Alam S., Anjum L., Haneef M. (2024). Investigating Non-steroidal Anti-Inflammatory Drugs as Potential Cancer Adjuncts: A Novel Approach to Cancer Therapy. RADS J. Biol. Res. Appl. Sci..

[30] Hu J., Qin S., Xiang Y., He Y., Hu H. (2023). Therapeutic potential of meloxicam in ameliorating sepsis-induced renal injury through the PI3K/AKT pathway. Sci. Adv. Mater..

[31] Cui L., Duan J., Mao P., Zhong J., He S., Dong J., Liu K., Guo L., Li J., Wang H. (2025). Meloxicam Alleviates Oxidative Stress Through Nrf2/HO-1 Activation in Bovine Endometrial Epithelial Cells. Vet. Sci..

[32] Cheng J., Liu Y., Mo L., Zeng F., Na Z., Yang B., Li M., Liu N. (2026). UBE2C/PTTG1 Regulates PANoptosis, Ferroptosis, and Autophagy to Improve Renal Ischemia–Reperfusion Injury. FASEB J..

[33] Cheng L., Hu Z., Gu J., Li Q., Liu J., Liu M., Li J., Bi X. (2024). Exploring COX-independent pathways: A novel approach for meloxicam and other NSAIDs in cancer and cardiovascular disease treatment. Pharmaceuticals.

[34] Asmarani Y.K., Haniastuti T. (2024). Analysis of Burkitt’s Lymphoma Cell Apoptosis After Treatment with Meloxicam: An In vitro Study using TUNEL Assay. Malays. J. Med. Health Sci..

[35] Trujillo-Rangel W.Á., García-Valdés L., Méndez-del Villar M., Castañeda-Arellano R., Totsuka-Sutto S.E., García-Benavides L. (2022). Therapeutic targets for regulating oxidative damage induced by ischemia-reperfusion injury: A study from a pharmacological perspective. Oxid. Med. Cell. Longev..

[36] Wu L., Xiong X., Wu X., Ye Y., Jian Z., Zhi Z., Gu L. (2020). Targeting oxidative stress and inflammation to prevent ischemia-reperfusion injury. Front. Mol. Neurosci..

[37] Conti P., Ronconi G., Caraffa A., Gallenga C., Ross R., Frydas I., Kritas S. (2020). Induction of pro-inflammatory cytokines (IL-1 and IL-6) and lung inflammation by Coronavirus-19 (COVI-19 or SARS-CoV-2): Anti-inflammatory strategies. J. Biol. Regul. Homeost. Agents.

[38] Khan J., Noboru N., Young A., Thomas D. (2017). Pro and anti-inflammatory cytokine levels (TNF-α, IL-1β, IL-6 and IL-10) in rat model of neuroma. Pathophysiology.

[39] Wautier J.-L., Wautier M.-P. (2023). Pro-and anti-inflammatory prostaglandins and cytokines in humans: A mini review. Int. J. Mol. Sci..

[40] Kirkby N.S., Sampaio W., Etelvino G., Alves D.T., Anders K.L., Temponi R., Shala F., Nair A.S., Ahmetaj-Shala B., Jiao J. (2018). Cyclooxygenase-2 selectively controls renal blood flow through a novel PPARβ/δ-dependent vasodilator pathway. Hypertension.

[41] Pefanis A., McRae J., Bongoni A., Ierino F., Cowan P. (2022). 422.7: Necroptosis plays a role in acute kidney injury (AKI) and the progression to renal fibrosis following ischemia reperfusion injury (IRI). Transplantation.

[42] Li S., Zhang W., Hu X. (2023). Comprehensive analysis of necroptosis-related genes in renal ischemia-reperfusion injury. Front. Immunol..

[43] Kolbrink B., von Samson-Himmelstjerna F.A., Murphy J.M., Krautwald S. (2023). Role of necroptosis in kidney health and disease. Nat. Rev. Nephrol..

[44] Liu X., Xie X., Ren Y., Shao Z., Zhang N., Li L., Ding X., Zhang L. (2021). The role of necroptosis in disease and treatment. MedComm.

[45] Pefanis A., Bongoni A.K., McRae J.L., Salvaris E.J., Fisicaro N., Murphy J.M., Ierino F.L., Cowan P.J. (2023). Dynamics of necroptosis in kidney ischemia-reperfusion injury. Front. Immunol..

[46] Dai Q., Zhang Y., Liao X., Jiang Y., Lv X., Yuan X., Meng J., Xie Y., Peng Z., Yuan Q. (2020). Fluorofenidone alleviates renal fibrosis by inhibiting necroptosis through RIPK3/MLKL pathway. Front. Pharmacol..

[47] Han Z., Luo Y., Chen H., Zhang G., You L., Zhang M., Lin Y., Yuan L., Zhou S. (2024). A deep insight into ferroptosis in renal disease: Facts and perspectives. Kidney Dis..

[48] Tang Z., Wang Y., Liu Y., Li C. (2023). Salidroside inhibits renal ischemia/reperfusion injury-induced ferroptosis by the PI3K/AKT signaling pathway. Exp. Ther. Med..

[49] Martin-Vega A., Cobb M.H. (2023). Navigating the ERK1/2 MAPK cascade. Biomolecules.

[50] Hassan M., Aboelnaga M.M., Al-Arman M., Hatata E.Z. (2021). Urinary cystatin C as a biomarker of early renal dysfunction in type 2 diabetic patients. Diabetes Metab. Syndr. Clin. Res. Rev..

[51] Çakır M., Aydın A., Fırat S., Şekerci G., Bircan B., Öz S. (2025). Protective effect of transient receptor potential ankyrin 1 inhibition on renal ischemia reperfusion injury in rats. J. Biochem. Mol. Toxicol..

[52] Chen J., Tang T.-T., Cao J.-Y., Li Z.-L., Zhong X., Wen Y., Shen A.-R., Liu B.-C., Lv L.-L. (2023). KIM-1 augments hypoxia-induced tubulointerstitial inflammation through uptake of small extracellular vesicles by tubular epithelial cells. Mol. Ther..

[53] Stehlé T., Delanaye P. (2024). Which is the best glomerular filtration marker: Creatinine, cystatin C or both?. Eur. J. Clin. Investig..

[54] Pottel H., Delanaye P., Cavalier E. (2024). Exploring renal function assessment: Creatinine, cystatin C, and estimated glomerular filtration rate focused on the European Kidney Function Consortium Equation. Ann. Lab. Med..

[55] Sari F.T., Sari F., Sari F., Arfian N., Sari D. (2020). Effect of kidney ischemia/reperfusion injury on proliferation, apoptosis, and cellular senescence in acute kidney injury in mice. Med. J. Malays..

[56] Afolabi O.A., Akhigbe T.M., Hammed S.O., Hamed M.A., Ekundina V.O., Ajike R.A., Alabi B.A., Akhigbe R.E. (2024). Moringa oleifera-based feed supplement protects against renal ischaemia/reperfusion injury via downregulation of Bax/caspase 3 signaling. Front. Nutr..

[57] Kari S., Subramanian K., Altomonte I.A., Murugesan A., Yli-Harja O., Kandhavelu M. (2022). Programmed cell death detection methods: A systematic review and a categorical comparison. Apoptosis.

[58] Bock F.J., Riley J.S. (2023). When cell death goes wrong: Inflammatory outcomes of failed apoptosis and mitotic cell death. Cell Death Differ..

[59] Han D.S., Ni M., Chan R.W., Chan V.W., Lui K.O., Chiu R.W., Lo Y.D. (2020). The biology of cell-free DNA fragmentation and the roles of DNASE1, DNASE1L3, and DFFB. Am. J. Hum. Genet..

[60] An Y., Zhao X., Zhang Z., Xia Z., Yang M., Ma L., Zhao Y., Xu G., Du S., Wu X.A. (2023). DNA methylation analysis explores the molecular basis of plasma cell-free DNA fragmentation. Nat. Commun..

[61] Tolba R.H., Fet N., Yonezawa K., Taura K., Nakajima A., Hata K., Okamura Y., Uchinami H., Klinge U., Minor T. (2014). Role of preferential cyclooxygenase-2 inhibition by meloxicam in ischemia/reperfusion injury of the rat liver. Eur. Surg. Res..

[62] Khan H., Sharma K., Kumar A., Kaur A., Singh T.G. (2022). Therapeutic implications of cyclooxygenase (COX) inhibitors in ischemic injury. Inflamm. Res..

